# SOD1 regulates ribosome biogenesis in KRAS mutant non-small cell lung cancer

**DOI:** 10.1038/s41467-021-22480-x

**Published:** 2021-04-15

**Authors:** Xiaowen Wang, Hong Zhang, Russell Sapio, Jun Yang, Justin Wong, Xin Zhang, Jessie Y. Guo, Sharon Pine, Holly Van Remmen, Hong Li, Eileen White, Chen Liu, Megerditch Kiledjian, Dimitri G. Pestov, X. F. Steven Zheng

**Affiliations:** 1 Rutgers Cancer Institute of New Jersey, Rutgers, The State University of New Jersey, New Brunswick, NJ USA; 2 Department of Pharmacology, Robert Wood Johnson Medical School, Rutgers, The State University of New Jersey, Piscataway, NJ USA; 3 Graduate Program in Cellular and Molecular Pharmacology, Rutgers, The State University of New Jersey, Piscataway, NJ USA; 4 Department of Cell Biology and Neuroscience, School of Osteopathic Medicine, Rowan University, Stratford, NJ USA; 5 Department of Cell Biology and Neuroscience, Rutgers, The State University of New Jersey, Piscataway, NJ USA; 6 Department of Medicine, Robert Wood Johnson Medical School, Rutgers, The State University of New Jersey, New Brunswick, NJ USA; 7 Aging and Metabolism Program, Oklahoma Medical Research Foundation, Oklahoma City, OK USA; 8 Center for Advanced Proteomics Research and Department of Microbiology, Biochemistry and Molecular Genetics, New Jersey Medical School, Rutgers, The State University of New Jersey, Piscataway, NJ USA; 9 Department of Molecular Biology and Biochemistry, Rutgers, The State University of New Jersey, Piscataway, NJ USA; 10 Department of Pathology, Robert Wood Johnson Medical School, Rutgers, The State University of New Jersey, New Brunswick, NJ USA

**Keywords:** Cancer, Cancer models, Cancer therapy, Targeted therapies, Lung cancer

## Abstract

SOD1 is known as the major cytoplasmic superoxide dismutase and an anticancer target. However, the role of SOD1 in cancer is not fully understood. Herein we describe the generation of an inducible *Sod1* knockout in KRAS-driven NSCLC mouse model. *Sod1* knockout markedly reduces tumor burden in vivo and blocks growth of KRAS mutant NSCLC cells in vitro. Intriguingly, SOD1 is enriched in the nucleus and notably in the nucleolus of NSCLC cells. The nuclear and nucleolar, not cytoplasmic, form of SOD1 is essential for lung cancer cell proliferation. Moreover, SOD1 interacts with PeBoW complex and controls its assembly necessary for pre-60S ribosomal subunit maturation. Mechanistically, SOD1 regulates co-localization of PeBoW with and processing of pre-rRNA, and maturation of cytoplasmic 60S ribosomal subunits in KRAS mutant lung cancer cells. Collectively, our study unravels a nuclear SOD1 function essential for ribosome biogenesis and proliferation in KRAS-driven lung cancer.

## Abstract (web summary)

The superoxide dismutase SOD1 is highly expressed in lung cancer but its role has not fully investigated yet. In this study, the authors demonstrate that SOD1 regulates ribosome biogenesis driving KRAS-driven lung tumorigenesis.

## Introduction

Superoxide dismutase (SOD) is an antioxidant enzyme that catalyzes the dismutation of superoxide radicals to hydrogen peroxide and molecular oxygen. There are three SODs in mammals. SOD1 (Cu/Zn-SOD), the major SOD, is distributed throughout the cytosol, mitochondrial intermembrane space, and nucleus^1,2^. SOD2 (Mn-SOD) is localized exclusively in the mitochondrial matrix. SOD3 (EC-SOD), also a copper-zinc-containing SOD, is secreted into the extracellular space. SODs efficiently detoxify superoxide radicals generated by various cellular processes, particularly mitochondria, and are well known as the first line of defense in oxidative stress^3^. Growing evidence indicates that SOD1 is also involved in redox signaling to regulate growth and metabolic pathways^4^, including glucose metabolism and transcription^2,5,6^. For example, SOD1 represses respiration and promotes glycolysis in the presence of glucose and oxygen^6^, and SOD1 is found in the nucleus of yeast and mammalian cells, where it is associated with promoters and regulates transcription of stress response genes to counter elevated oxidative conditions^2^.

As a major antioxidant enzyme, SOD1 has been closely linked to cancer. Cancer cells tend to have a high reactive oxygen species (ROS) content due to aberrant energy metabolism. Cancer cells become increasingly dependent on activated antioxidants such as NRF2 to prevent excessive cellular damage and apoptosis during tumor progression. Consistently, overexpression of SOD1 is observed in lung^7^ and mammary tumors^8,9^. In vitro studies show that SOD1 is essential for the growth of non-small cell lung cancer (NSCLC) and leukemia as knockdown or pharmacological inhibition of SOD1 potently inhibits the growth of NSCLC cell lines driven by oncogenic KRAS and EGFR^7,10^, as well as other cancer cell lines and xenograft tumors^7,9,11^. Moreover, SOD1 deficiency blunts ERBB2-driven mammary tumorigenesis^8^. SOD1 has been recognized as a promising anticancer drug target with several small-molecule targeting agents identified and under preclinical and clinical development in recent years^4^. Despite this progress, the role of SOD1 in lung cancer has not been studied genetically in relevant animal models in vivo.

Lung cancer is the leading cause of cancer death worldwide^12^. Adenocarcinoma is diagnosed in 50% of lung cancer patients^13^. Mutant KRAS is a driver oncogene for ~30% of human lung adenocarcinoma (LUCA)^14^. Despite considerable efforts, therapeutic targeting of mutant KRAS has proven to be very difficult, posing a major challenge to effective lung cancer therapy. Multiple large-sample gene expression profiling studies of primary lung tumor tissues have revealed statistically significant increases in SOD1 expression in NSCLC^10^. Small-molecule inhibitors targeting SOD1 have been shown to effectively suppress the growth of lung cancer cells in vitro and xenograft tumors in vivo^7,10,15–17^.

In this work, we generated an inducible *Sod1* knockout in the KP model driven by oncogenic KRAS^G12D^ and *TP53* deletion. Our results provide in vivo genetic evidence for the critical role of SOD1 in lung cancer tumorigenesis. Our data further reveal that SOD1 is localized in the nucleus, which sustains cancer cell growth by promoting hyperactive ribosome biogenesis through PeBoW complex-dependent pre-ribosomal RNA (rRNA) processing and ribosome biogenesis in KRAS-driven NSCLC.

## Results

### Generation of tamoxifen (TAM)-inducible *Sod1* knockout in the KRAS-p53 (KP) NSCLC genetically engineered mouse (GEM) model

**Fig. 1:**
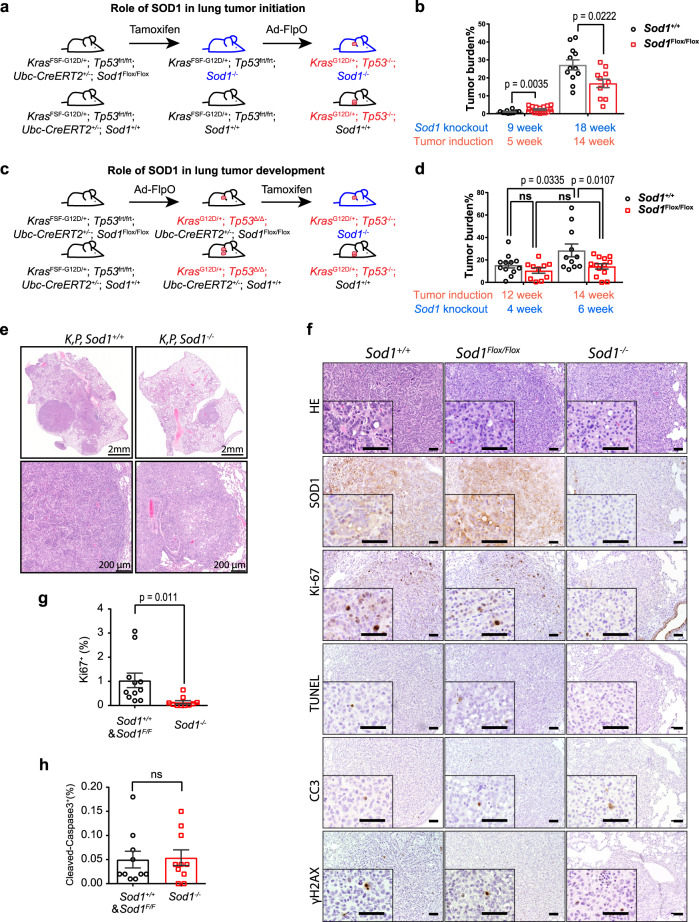
SOD1 is required for KRas-p53 (KP) lung cancer development/maintenance. **a** Experimental scheme for examining the role of SOD1 in KP lung tumor initiation in mice. Blue color indicates *Sod1* knockout and red color indicates KP activated. **b** Quantification of lung tumor burden in *Sod1*^+/+^ KP (*n* = 10 for 5-week groups and *n* = 12 for 14-week groups) and *Sod1*^−/−^ KP (*n* = 16 for 5-week groups, and *n* = 11 for 14-week groups) following tumor induction (administration of adenovirus expressing FlpO (Ad-FlpO)), and 14 and 19 weeks post *Sod1* knockout (administration of tamoxifen (TAM)). Data are shown as mean ± SEM (standard error of the mean). Statistical significance was tested using two-tailed unpaired *t* test, *p* = 0.0035 (tumor induction in 5-week groups), *p* = 0.0222 (tumor induction in 14-week groups). **c** Experimental scheme for examining the role of SOD1 in KP lung tumor maintenance. **d** Quantification of tumor burden in *Sod1*^+/+^ KP (*n* = 12 for 12-week groups and *n* = 10 for 14-week groups) and *Sod1*^−/−^ *KP* (*n* = 11 for 12-week groups and *n* = 13 for 14-week groups) post tumor induction (Ad-FlpO administration), and 4-week and 6-week post TAM. Data are shown as mean ± SEM. Statistical significance was tested using two-tailed unpaired *t* test. n.s., not significant. *p* = 0.0335 (tumor induction 12-week group), *p* = 0.0107 (tumor induction 14 weeks group). **e** Representative images of H&E (hematoxylin and eosin)-stained lung sections from *Sod1*^+/+^ KP and *Sod1*^−/−^ KP mice. *n* = 10 mice per mouse group. Representative data from each group are shown. **f** Representative H&E staining and IHC staining for SOD1, Ki-67, TUNEL, CC3, and γH2AX in lung tumor sections from 4OHT (4-hydroxytamoxifen)-treated *Sod1*^+/+^ or *Sod1*^*Flox/Flox*^ mice bearing KP tumors. Scale bar, 100 μm. *Sod1*^*Flox/Flox*^ tumors are those occurring in *Sod1*^*Flox/Flox*^ mice that have escaped Cre/LoxP recombination as indicated by SOD1-positive staining. *Sod1*^−/−^ tumors are those with *Sod1* knockout. *n* = 3 mice per mouse group. Representative data from each group are shown. **g** Quantification of Ki-67-positive cells in lung tumors with or without SOD1 (*n* = 11 for *Sod1*^*Flox/Flox*^ and the *Sod1*^+/+^ group and *n* = 10 for the *Sod1*^Δ/Δ^ group). *Sod1*^*Flox/Flox*^ tumors are counted as part of the *Sod1*^+/+^ group. Data are shown as mean ± SEM. Statistical significance was tested using two-tailed unpaired *t* test. **h** Quantification of cleaved caspase-3-positive cells in lung tumors with or without SOD1 (*n* = 10 for *Sod1*^*Flox/Flox*^ and the *Sod1*^+/+^ group, *n* = 10 for the *Sod1*^Δ/Δ^ group). Data are shown as mean ± SEM. Statistical significance was tested using two-tailed unpaired *t* test. ns, not significant.

Knockdown or pharmacological inhibition of SOD1 has been shown to effectively suppress the growth and proliferation of lung cancer cells and xenograft tumors^7,10,15–17^. However, the role of SOD1 in lung cancer has not been investigated genetically. We, therefore, generated TAM-inducible whole-body *Sod1* knockout in the KP lung cancer GEM model^18^ for studying the role of SOD1 in lung tumor initiation and development (see Fig. 1a and Supplementary Fig. 1a for experimental schemes). In the KP model, lung tumors can be initiated by intranasal administration of adenovirus expressing the FLP DNA recombinase (Ad-FLPo)^18^. Floxed *Sod1* mice (*Sod1*^Flox/Flox^)^19^, UBC-Cre-ERT2 mice^20^, *Kras*^FSF-G12D/+^ mice (JAX stock #008653), and Trp5^frt/frt^ mice (JAX stock #017767) were bred to give rise to *Sod1*^Flox/Flox^; UBC-Cre-ERT2; *Kras*^FSF-G12D/+^; *Trp5*^*frt/frt*^ (for inducing lung tumors and *Sod1* knockout) or *Sod1*^Flox/Flox^; *Kras*^FSF-G12D/+^; *Trp5*^*frt/frt*^ (for inducing lung tumors but no *Sod1* knockout) mice. TAM treatment induced efficient *Sod1* knockout (*Sod1*^−/−^) that was verified by PCR genotyping (Supplementary Fig. 1b) and by Western blot (WB) (Supplementary Fig. 1c). Inducible knockout of *Sod1* at the age of 6–8 weeks caused a slight reduction in body weight during the first 9 months of age (Supplementary Fig. 2a). Otherwise, no apparent histological abnormality was observed with *Sod1*^−/−^ mice by tissue hematoxylin and eosin (H&E) staining at 5 months post TAM (Supplementary Fig. 2b and data not shown) except for mild liver steatosis (Supplementary Fig. 2c).

### SOD1 is required for mouse KP lung tumor development and maintenance

To study the role of SOD1 in lung tumor initiation, we used TAM to first induce *Sod1* knockout in *Sod1*^*Flox/Flox*^, KP mice at ~8 weeks old, and 4 weeks later, lung tumorigenesis was induced by intranasal administration of adenovirus expressing FlpO (Ad-FlpO) (Fig. 1a and Supplementary Fig. 1a, top panel). Adenomas started to appear by 5 weeks post tumor initiation. At this time point, *Sod1*^−/−^ KP mice showed a slightly higher lung tumor burden than *Sod1*^+/+^ KP mice (Fig. 1b), indicating a mild effect of SOD1 deficiency in tumorigenesis. However, by 14 weeks post tumor initiation when adenocarcinomas were developed, *Sod1*^−/−^ KP mice showed significantly less tumor burden than *Sod1*^+/+^ KP mice (Fig. 1b), suggesting that SOD1 is required for KP tumor development and/or maintenance. To ask if SOD1 is required for lung tumor development and maintenance, lung tumors were first initiated with nasal Ad-FlpO administration in ~8-week-old *Sod1*^*Flox/Flox*^ mice, which was followed by TAM injection 10 weeks later to knock out *Sod1* (Fig. 1c and Supplementary Fig. 1a, lower panel). At 6 weeks post *Sod1* knockout (TAM), tumor burden in *Sod1*^−/−^ KP mice was analyzed and found significantly lower than that in *Sod1*^+/+^ KP mice (Fig. 1d, e). Comparing tumor burden between 4 and 6 weeks after *Sod1* knockout, the increase of tumor burden in *Sod1*^−/−^ KP mice was much smaller than that of *Sod1*^+/+^ KP (Fig. 1d). Consistently, *Sod1*-null tumors showed considerably lower Ki-67 staining than wild-type tumors (Fig. 1f, g). In contrast, loss of SOD1 did not significantly affect the level of apoptosis or DNA damage as indicated by caspase-3 cleavage/TUNEL (terminal deoxynucleotidyl transferase dUTP nick-end labeling) or γH2AX staining, respectively (Fig. 1f, h). Together, these results provide in vivo evidence that SOD1 is required for KP lung tumor development and maintenance by sustaining the oncogenic proliferative capacity.

### SOD1 is required for the growth of KRAS mutant NSCLC cells in vitro

**Fig. 2:**
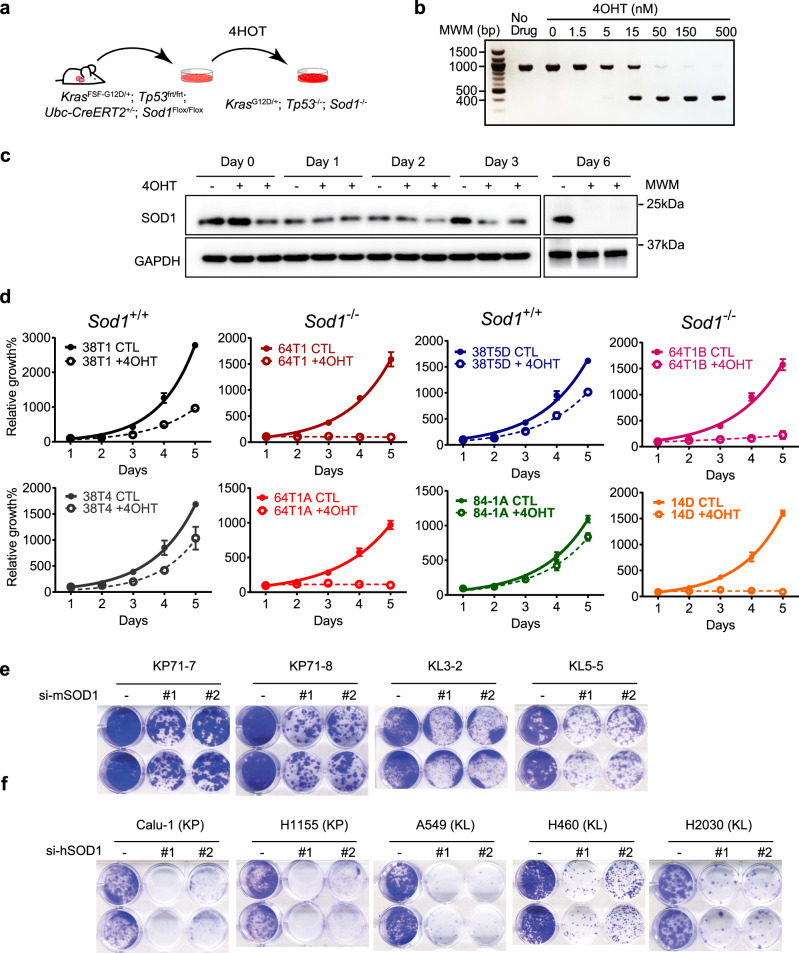
SOD1 is required for the proliferation of KRAS mutant NSCLC cells. **a** Experimental scheme to generate KP NSCLC cell lines and induce *Sod1* knockout by 4OHT. **b** Genotyping PCR analysis of genomic DNA from *Sod1*^*Flox/Flox*^ KP NSCLC cells following treatment with different doses of 4OHT. Data are represented by three independent experiments (marker unit: bp). **c** Western blot analysis of SOD1 protein in KP NSCLC cells at different days following treatment with 4OHT. Data are represented by three independent experiments. **d** SOD1 is required for the growth of KP NSCLC cells. Different *Sod1*^+/+^ and *Sod1*^*Flox/Flox*^ KP NSCLC cell clones (as numbered) were treated with 4OHT overnight, and then cultured for the indicated number of days. Cell growth was measured using the SRB assay. Tetrahydrofuran was used as vehicle control (CTL). Data are represented by means ± SD (standard deviation) of three independent experiments. **e** *Sod1* was knocked down by two independent siRNAs (si-mSOD1) in mouse KP and KL NSCLC cell lines. Cell growth was measured by plate growth assay. “−” is control siRNA. Shown are duplicates for each experiment. **f** SOD1 was knocked down by siRNA (si-hSOD1) in human KP and KL (KRas-Lkb1) NSCLC cell lines. Cell growth was measured by plate growth assay. “−” indicates control siRNA. Shown are duplicates for each experiment.

To investigate the molecular function of SOD1 in lung cancer cells, we derived NSCLC cell lines from *Sod1*^+/+^ or *Sod1*^*Flox/Flox*^ KP lung tumors (Fig. 2a). To induce *Sod1* knockout, mouse NCLSC cells were treated with (*Z*)-4-hydroxytamoxifen (4OHT) overnight, and then washed with a fresh medium. As low as 50 nM 4OHT achieved near complete deletion of the *Sod1* genomic locus, as evaluated by PCR genotyping (Fig. 2b). SOD1 is a stable protein with a long half-life^21^. It took ~6 days post 4OHT treatment for SOD1 protein to become undetectable by WB (Fig. 2c). Some growth inhibition by 4OHT treatment was observed in wild-type cells (Fig. 2d), suggesting that there is a mild toxicity associated with 4OHT, which is likely due to 4OHT inhibition of estrogen receptor (ER) that plays a role in lung cancer cell growth^22^. Therefore, *Sod1*^+/+^ KP cells were included in all assays as a control for the 4OHT effect. 4OHT treatment blocked the growth of *Sod1*^*Flox/Flox*^ KP cell clones, but only had a slight-to-moderate effect on *Sod1*^+/+^ KP cells (Fig. 2d). In addition to TP53, LKB1 mutation also frequently cooccurs with that of KRAS in human NSCLC tumors. To ask the role of SOD1 in different KRAS mutant lung cancer cells, we knocked down SOD1 in both KP and KRAS-LKB1 (KL) mouse and human NSCLC cell lines (Supplementary Fig. 3). The results showed that SOD1 knockdown attenuated the growth of mouse and human KP and KL lung cancer cells (Fig. 2e, f). These results indicate that SOD1 is generally required for the growth of KRAS mutant NSCLC cells rather than just for KP cells.

### SOD1 knockout/knockdown does not significantly alter ROS level in KRAS mutant NSCLC cells

Because SOD1 is a major antioxidant enzyme, we anticipated that *Sod1* knockout would cause elevated ROS, which would be responsible for the inhibition of KP cell proliferation. Unexpectedly, loss of SOD1 did not significantly affect superoxide and peroxide levels in KP cells under normal culture conditions, as detected by dihydroethidium (DHE) (Supplementary Fig. 4a). Similarly, loss of SOD1 only marginally increased general ROS levels in KP cells, as measured by CM-H_2_DCFDA under basal conditions (Supplementary Fig. 4b). To ask if *Sod1* knockout affects the cellular response to oxidative stress, we treated KP cells with paraquat to elicit mitochondrial superoxide production. Paraquat caused a marked increase in superoxide level in *Sod1* knockout cells but not in the wild-type cells (Supplementary Fig. 4a), while H_2_O_2_ increased general ROS levels regardless of *Sod1* status (Supplementary Fig. 4b). Similar results were observed with SOD1 knockdown mouse and human KP and KL NSCLC cell lines (Supplementary Fig. 5). These results demonstrate that SOD1 plays a crucial, specific role in superoxide defense only under oxidative stress conditions, but is dispensable under normal conditions.

To ask if the growth defect of *Sod1*^−/−^ KP cells is due to impaired ROS regulation, we treated these cells with different chemical antioxidants. *N*-acetyl-l-cysteine is a commonly used thiol antioxidant, but failed to relieve the growth inhibition from *Sod1* knockout (Supplementary Fig. 4c, d). Similarly, a diverse group of other commonly used antioxidants was also unable to restore growth of *Sod1*^−/−^ KP cells, including MnTBAP, a cell-permeable SOD mimetic and peroxynitrite scavenger (Supplementary Fig. 4e); Tempol, a SOD mimetic with the ability to degrade superoxide radicals (Supplementary Fig. 4f); GSH-MEE (glutathione reduced ethyl ester), a membrane/lipid permeable derivative of GSH (Supplementary Fig. 4g); and Trolox (6-hydroxy-2,5,7,8-tetramethyl-chromane-2-carboxylic acid), a water-soluble analog of vitamin E (Supplementary Fig. 4h). NRF2 is a transcription factor that is activated in cells in response to oxidative stress, leading to upregulation of NRF2 target genes such as *Nqo1*^23,24^. However, the expression of Nqo1 transcription remained largely unchanged in *Sod1*^−/−^ KP cells compared with *Sod1*^+/+^ KP cells (Supplementary Fig. 4i). Similar results were seen with human KP and KL NSCLC cells (Supplementary Fig. 6). Moreover, *Sod1* knockout did not significantly alter the phosphorylation of EGFR, p38 MAPK, and ERK (Supplementary Fig. 7), as previously suggested with copper chelating by ATN-224 through ROS-mediated mechanisms^5,7^. Collectively, these results indicate that *Sod1* knockout does not significantly affect superoxide or ROS levels. They further suggest that an ROS-independent function of SOD1 is likely responsible for the growth defect in KP cells.

### Nuclear SOD1 is essential for the proliferation of KRAS mutant NSCLC cells

**Fig. 3:**
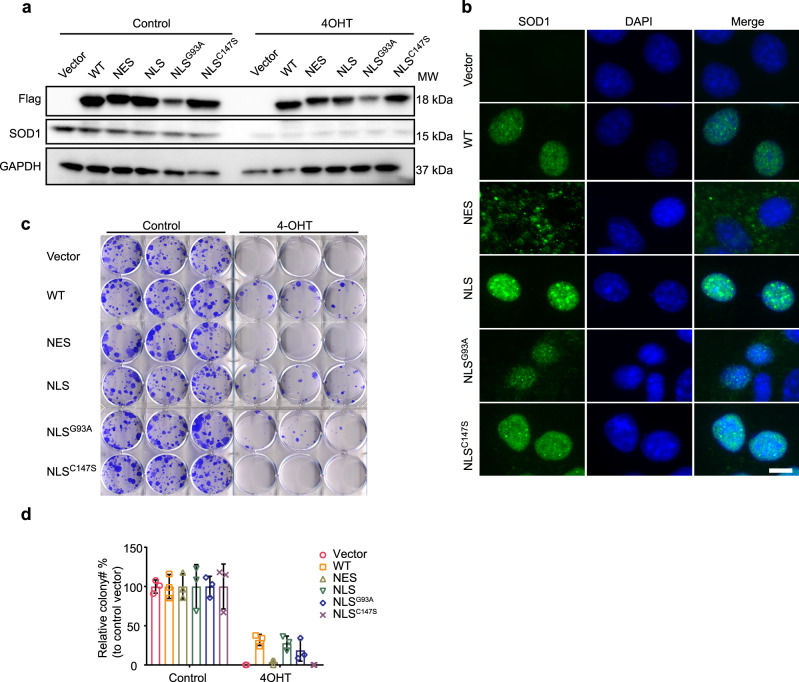
Nuclear SOD1 is necessary and sufficient to support KP NSCLC cell proliferation. **a**, **b** Flag-SOD1 tagged with an NLS or NES was transiently expressed in *Sod1*^*Flox/Flox*^ KP NSCLC cells treated with 4OHT, and cells were analyzed by Western blot (**a**) and IF staining (**b**) (scale bar, 10 μm). Vector, vector control; WT, wild type SOD1; NES, SOD1-NES; NLS, SOD1-NLS; NLS^G93A^, SOD1^G93A^-NLS; NLS^C147S^, SOD1^C147S^-NLS. Data are represented by three independent experiments. **c** Clonogenic proliferation assay of *Sod1*^*Flox/Flox*^ KP cells treated with or without 4OHT overnight, followed by transient expression of different tagged Flag-SOD1. Cells were cultured for 10 days before staining with crystal violet. **d** Quantification of the colony formation results in (**c**). Data are represented as means ± SD of three independent experiments.

Although SOD1 was thought to be mainly a cytoplasmic enzyme^1^, recent studies revealed that SOD1 is also localized in the nucleus^2^. Consistently, SOD1 was also found in the nuclei of human immortalized airway epithelial cells (Beas2B) and KP and KL NSCLC cells (Supplementary Fig. 7). To ask if SOD1 nuclear localization plays a role in the proliferation of KP NSCLC cells, SOD1 was tagged with an SV40 nuclear localization sequence (NLS, “PKKKRKV”) or a nuclear export sequence (NES, “LALTHILVLHYGL”). Untagged or tagged SOD1 was transiently expressed in *Sod1*^*Flox/Flox*^ KP cells (Fig. 3a). 4OHT was then added to induce knockout of endogenous *Sod1*, allowing assay of the functionality of tagged proteins. SOD1-NES and SOD1-NLS were exclusively cytoplasmic and nuclear, respectively, indicating that the tags targeted SOD1 to the proper subcellular locations (Fig. 3b). Interestingly, untagged SOD1 was also largely nuclear (Fig. 3b). Flag-SOD1 was transiently transfected in ~10–20% of cells (data not shown), explaining the growth restoration of *Sod1*^−/−^ KP NSCLC cells by Flag-SOD1 at ~10–20% efficiency in the colony formation assay (Fig. 3c, d). Remarkably, Flag-SOD1-NLS, but not Flag-SOD1-NES, was also able to suppress the growth defect of the *Sod1* knockout (Fig. 3c, d). C147 is critical for the enzymatic activity of SOD1, and the C147S mutant is catalytically inactive^25^. Strikingly, Flag-SOD1^C147S^-NLS lost the ability to suppress the growth defect of the *Sod1* knockout (Fig. 3c, d), suggesting that the enzymatic activity is important for nuclear SOD1 function to support proliferation. G93A is an amyotrophic lateral sclerosis (ALS)-associated mutation that does not affect SOD1’s enzymatic activity^21,26,27^. Interestingly, Flag-SOD1^G93A^ retained the ability to suppress the growth defect of *Sod1* knockout cells (Fig. 3c, d). Taken together, these results indicate that nuclear SOD1 plays a critical role in the proliferation of KP cells, which is dependent on its enzymatic activity.

### SOD1 interacts with and regulates the assembly of the PeBoW complex

**Fig. 4:**
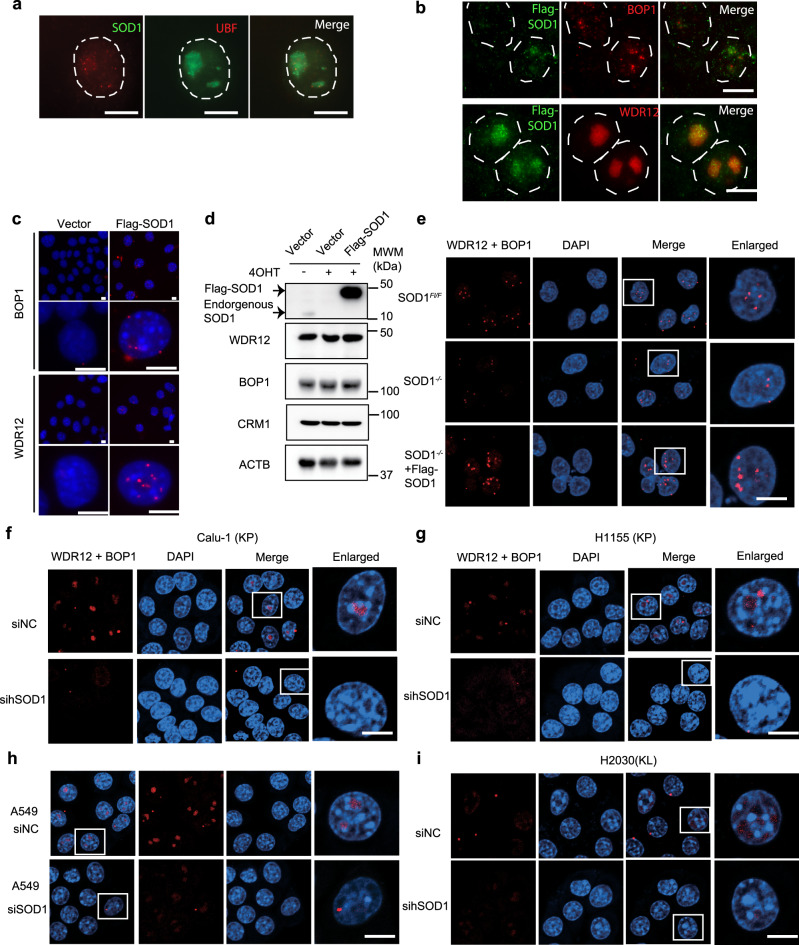
SOD1 binds to BOP1 and WDR12, and regulates the assembly of PeBoW complex. **a** Flag-SOD1 is prominently localized in the nucleoli. *Sod1*^*Flox/Flox*^ KP NSCLC cells stably expressing Flag-SOD1 were treated with 4OHT and doxycycline was added to the medium to induce expression of Flag-SOD1. Cells were fixed on day 5 post treatment and analyzed by immunofluorescence analysis using antibodies against Flag (red) and UBF (green, nucleoli). Dashed lines demarcate the nucleus. Scale bars represent 10 μm. Data are represented by three independent experiments. **b** Flag-SOD1 is colocalized with BOP1 and WDR12. *Sod1*^*Flox/Flox*^ KP cells stably expressing Flag-SOD1 were treated with 4OHT and doxycycline to induce knockout of endogenous *Sod1* and expression of ectopic Flag-SOD1, respectively. IF analysis was performed with antibodies against Flag (green) and BOP1 or WDR12 (red). Dashed lines indicate the nucleus. Scale bar represents 10 μm. Data are represented by three independent experiments. **c** SOD1 interacts with the PeBoW complex in intact cells as judged by Duolink. *Sod1*^*Flox/Flox*^ KP cells stably expressing Flag-SOD1 were treated with 4OHT and doxycycline to induce knockout of endogenous *Sod1* and expression of ectopic Flag-SOD1, respectively. Cells were fixed and analyzed by Duolink to detect the interaction of Flag-SOD1 and WDR12 or BOP1 (red dots). Shown is a representative image. Scale bars, 10 μm. Data are represented by three independent experiments. **d** *Sod1* knockout or overexpression does not affect the protein level of CRM1, BOP1, and WDR12. Same as (**c**) except that samples were analyzed by immunoblot. Data are represented by three independent experiments. **e** *Sod1* knockout disrupts the interaction between BOP1 and WDR12 in mouse KP NSCLC cells. Mouse *Sod1*^*Flox/Flox*^ KP NSCLC cells were treated with or without 4OHT to knock out endogenous *Sod1*, and Dox to induced Flag-SOD1 expression. BOP1–WDR12 interaction was analyzed by Duolink. Scale bar represents 10 μm. Data are represented by three independent experiments. **f**–**i** SOD1 knockdown disrupts the interaction between BOP1 and WDR12 in human KP and KL NSCLC cells. Human KP and KL NSCLC cells were transfected with SOD1 human siRNA (si-hSOD1) or a control siRNA (siNC). BOP1–WDR12 interaction was analyzed by Duolink. Scale bar represents 10 μm. Data are represented by three independent experiments.

To gain an insight into the function of SOD1 in the nucleus, we transiently expressed Flag-SOD1 in *Sod1*^−/−^ NSCLC cells and immunoprecipitated Flag-SOD1 using anti-Flag antibody. Liquid chromatography-tandem mass spectrometry (LC-MS/MS) analysis identified BOP1 and WDR12 as SOD1-binding proteins. BOP1 and WDR12 are components of the PeBoW (PES1-BOP1-WDR12) heterotrimeric complex essential for the processing of pre-rRNA and maturation of the 60S ribosomal subunit^28,29^. Interestingly, PeBoW proteins are overexpressed in cancer, enhancing ribosome biogenesis and cancer cell growth, suggesting that PeBoW is an oncogenic target for promoting ribosome biogenesis to support uncontrolled growth^30,31^. To verify the LC-MS/MS result, we performed immunofluorescence (IF) staining. The result showed that SOD1 was prominently localized in the nucleoli and was colocalized with BOP1 and WDR12 (Figs. 4a, b and Supplementary Fig. 8). We further found that SOD1 interacts with BOP1 and WDR12 in NSCLC cells as detected by Duolink, a method that detects protein–protein interactions in situ in intact cells^32^ (Fig. 4c, d). Assembly of the PeBoW complex is known to be essential for ribosome biogenesis and cell growth^33^. We, therefore asked if SOD1 regulates the formation of the PeBoW complex. In control KP mouse NSCLC cells, BOP1–WDR12 interaction was readily detectable by Duolink in the nucleus (Fig. 4e). However, their interaction was drastically reduced in SOD1^−/−^ cells (Fig. 4e). Overexpression of Flag-SOD1 in SOD1^−/−^ cells not only restored but also further enhanced BOP1–WDR12 interaction (Fig. 4e). Similarly, SOD1 knockdown drastically decreased BOP1–WDR12 interaction in KP and KL human NSCLC cell lines (Fig. 4f–i). These results show that SOD1 interacts with and regulates the stability of the PeBoW complex.

### SOD1 regulates pre-rRNA processing

**Fig. 5:**
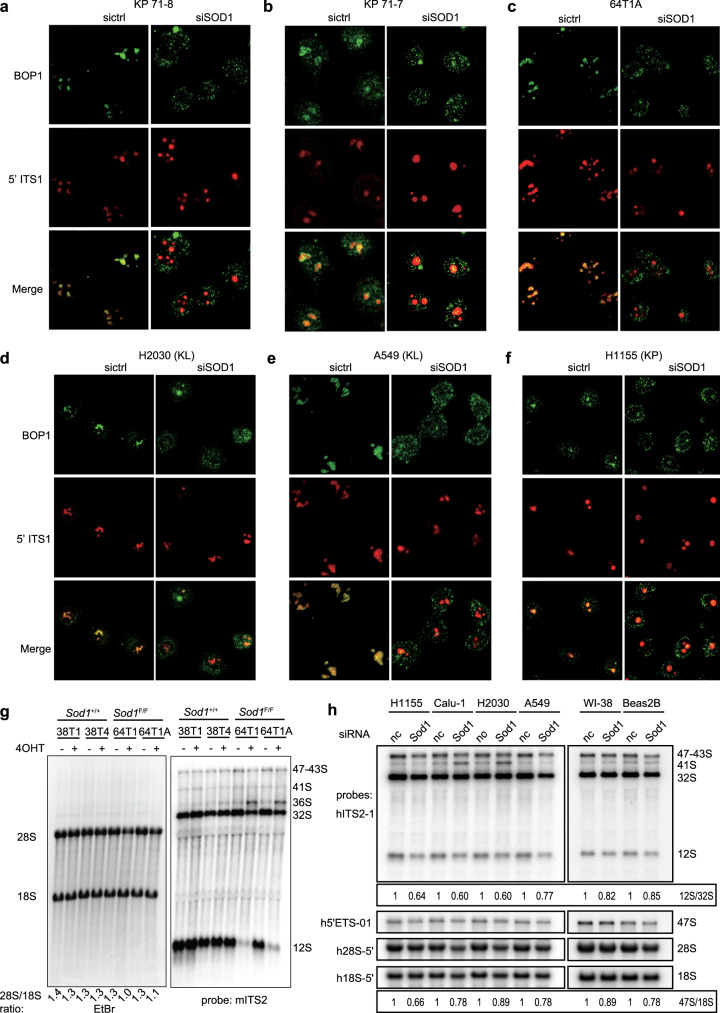
SOD1 regulates pre-rRNA processing in mouse and human KRAS mutant NSCLC cells. **a**–**c** SOD1 knockdown causes delocalization of BOP1 from pre-rRNA. Mouse NSCLC cells were transfected with mouse SOD1 siRNA (siSOD1) or a control siRNA (siNC). BOP1 and pre-rRNA were stained by IF and RNA-FISH, respectively. Scale bar represents 10 μm. Data are represented by three independent experiments. **d**–**f** SOD1 knockdown causes delocalization of BOP1 from pre-rRNA. Human KP and KL NSCLC cells were transfected with human SOD1 siRNA (siSOD1) or a control siRNA (siNC). BOP1 and pre-rRNA were stained by IF and RNA-FISH, respectively. Scale bar represents 10 μm. Data are represented by three independent experiments. **g** *Sod1* knockout impairs pre-rRNA processing. Total RNA was isolated from *Sod1*^*Flox/Flox*^ and *Sod1*^+/+^ KP cells treated with or without 4OHT. Total RNA separated by agarose gel was transferred to a membrane that was stained for 18S/28S total rRNAs by ethidium bromide (EtBr, left panel) and hybridized with a radiolabeled mouse mITS2 probe (right panel). **h** SOD1 knockdown impairs pre-rRNA processing. Total RNA was isolated from human NSCLC cells (KP: H1155, Calu-1; KL: H2030, A549), human normal lung fibroblasts (WI38), and human normal lung epithelial cells (Beas2B) transfected with human SOD1 siRNA (*Sod1*) or a control siRNA (nc). RNA separated by agarose gel was hybridized with radiolabeled human probes specific for the human 5′-ETS and ITS2 regions of pre-rRNAs, and 5′-regions of mature 28S and 18S rRNAs. The ratio for 12S/32S and 47S/18S is shown.

PeBoW complex is involved in the processing of pre-rRNA during nucleolar assembly and maturation of the 60S ribosomal subunit^28,29^. To gain an insight into how SOD1 controls PeBoW function, we first analyzed the localization of BOP1 by IF staining using a BOP1 antibody, in combination with RNA fluorescence in situ hybridization to visualize pre-rRNA with a probe specific for the internal transcribed spacer 1 (ITS1) of the pre-rRNA transcript (Supplementary Fig. 9). Consistent with the role of the PeBoW complex in pre-rRNA processing, BOP1 staining colocalized with the ITS probe signal in mouse and human KP and KL NSCLC cell lines (Fig. 5a–f). Conversely, BOP1 became dispersed throughout the nucleoplasm, delocalizing from the sites of pre-rRNA processing, after SOD1 knockdown (Fig. 5a–f). This result suggests that the association of the PeBoW complex with pre-rRNA is dependent on SOD1 in these NSCLC cells.

To further investigate pre-rRNA processing, we used a radiolabeled ITS2 probe (Supplementary Fig. 9)^34^ in a Northern blot analysis. Compared with *Sod1*^+/+^ KP NSCLC cells, 12S pre-rRNA was strongly depleted, while its precursor 32S pre-RNA showed a moderate reduction in *Sod1*^−/−^ KP NSCLC cells (Fig. 5g), mimicking pre-rRNA processing defects observed upon inhibition of PeBoW function through knockdowns and dominant-negative mutants of BOP1 and PES1^35,36^. Similar effects on pre-rRNA processing were observed after SOD1 knockdown in human KP and KL NSCLC cell lines (Fig. 5h). The negative impact of SOD1 knockdown on rRNA processing and cell proliferation was less pronounced in human normal lung fibroblast and epithelial cell lines (Fig. 5h and Supplementary Fig. 10a, b). The impairment of rRNA processing by SOD1 knockdown in human NSCLC cells is consistent with our in vivo data in the mouse showing that SOD1 knockout attenuated NSCLC tumor growth but did not cause any apparent abnormality in normal lungs. In addition, *Sod1*^−/−^ mouse cells accumulated 36S pre-rRNA, while human cells accumulated 41S pre-rRNA, the processing intermediates derived from an altered order of pre-rRNA cleavages occurring when pre-60S maturation is perturbed^34,36^. Of note, the 47S pre-rRNA/18S rRNA ratio was also decreased, suggesting that the rate of 47S pre-rRNA transcription is also negatively affected by SOD1 knockdown (Fig. 5h). Together, these results indicate that SOD1 regulates PeBoW complex-dependent processing of pre-rRNA during nucleolar maturation of the pre-60S ribosomal subunits.

### Nucleolar localization is sufficient for SOD1 to promote pre-rRNA processing and ribosome biogenesis

**Fig. 6:**
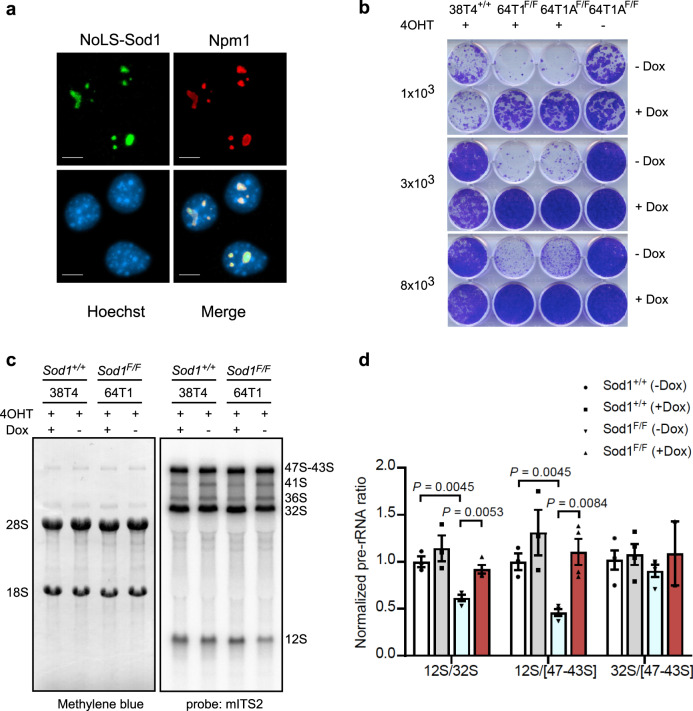
Nucleolar localization is sufficient for SOD1 to promote growth and pre-rRNA processing of NSCLC cells. **a** Dox-induced expression of NoLS-Sod1-Flag (NoLS-Sod1) in mouse *Sod1* knockout KP NSCLC cells. Mouse KP *Sod1*^*F/F*^ NSCLC cells were treated with 4OHT to knock out endogenous SOD1 in the absence or presence of Dox-induced NoLS-Sod1 expression. NoLS-Sod1-Flag was stained by IF with anti-Flag antibody. The nucleolus was stained by IF with an anti-Npm1 antibody, and the nucleus was stained with Hoescht 33342. A representative field from the analysis performed on 50–60 cells is shown. Scale bars, 10 μm. **b** NoLS-SOD1 is sufficient to suppress growth defects due to the knockout of endogenous *Sod1* in mouse KP NSCLC cells. Same as (**a**) except plate growth assay was performed. The number of cells plated per well is indicated on the left. **c** NoLS-SOD1 is sufficient to suppress pre-rRNA processing defects due to the knockout of endogenous *Sod1* in mouse KP NSCLC cells. Same as (**a**) except Northern blot analysis of pre-rRNAs was performed using a mouse ITS2 probe (right panel). Total 18S and 28S rRNAs were stained with methylene blue. **d** Quantification of results from (**c**). The ratios of 12S/32S and 12S/[47-43S] pre-rRNAs decreased by knockout of endogenous Sod1 are restored by expression of NoLS-SOD1. Bars show mean values ± SEM in biological replicates (*n* = 3 for *Sod1*^+/+^, *n* = 4 for *Sod1*^*F/F*^), significant differences are indicated (*p* < 0.01, unpaired two-tailed multiple *t* tests with Holm–Sidak correction).

Because SOD1 is found in the nucleolus and regulates pre-rRNA processing, we asked if its nucleolar localization is necessary for these SOD1 functions. To this end, we generated a doxycycline (Dox)-inducible nucleolar form of SOD1 by tagging it with a nucleolar localization sequence (NoLS-SOD1). After mouse KP *Sod1*^*Flox/Flox*^ NSCLC cells were treated with 4OHT to deplete endogenous SOD1, we used Dox to induce NoLS-SOD1 expression in the absence of endogenous SOD1 (Fig. 6a). As shown earlier, 4OHT treatment caused growth arrest of KP *Sod1*^*Flox/Flox*^ NSCLC cells (Fig. 6b). However, induction of NoLS-SOD1 expression with Dox rescued this growth defect (Fig. 6b). We next performed Northern blot analysis of pre-rRNAs, followed by the quantitative analysis of precursor levels^37^. In the absence of Dox induction of NoLS-SOD1, endogenous *Sod1* knockout impaired normal pre-rRNA processing, as indicated by the significantly diminished ratios of 12S/32S and 12S/[47-43S] pre-rRNAs (Fig. 6c, d). Remarkably, Dox induction of NoLS-SOD1 fully restored the normal ratios for these rRNA precursors (Fig. 6c, d). These results demonstrate that nucleolar localization of SOD1 is necessary and sufficient for promoting pre-rRNA processing and growth of these NSCLC cells.

### SOD1 regulates the biogenesis of 60S ribosomal subunits and nucleolar hypertrophy in NSCLC

**Fig. 7:**
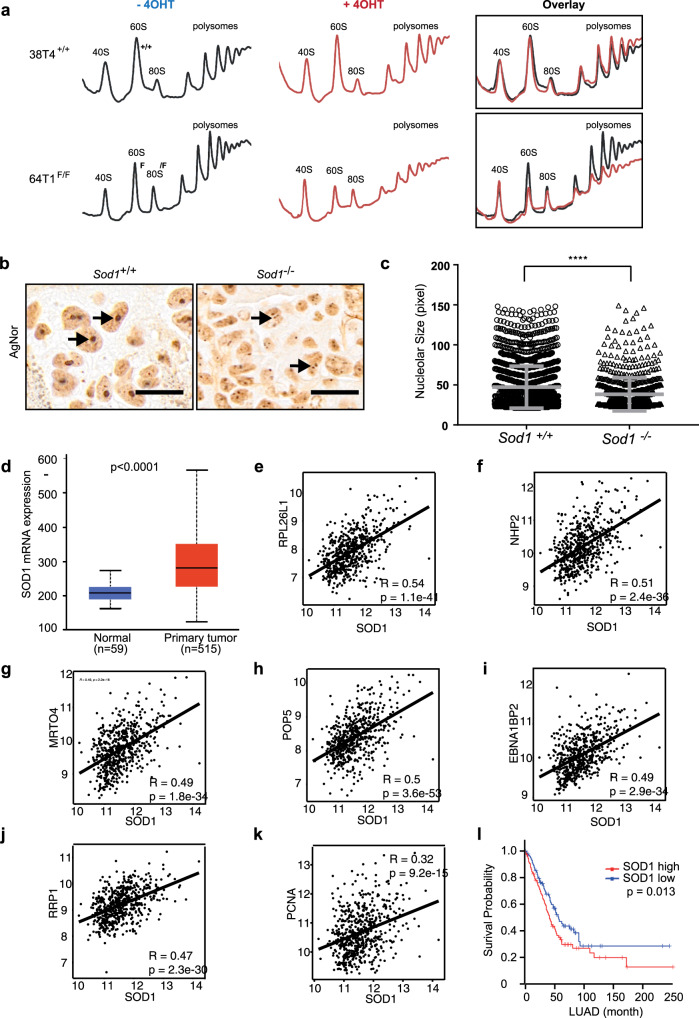
SOD1 promotes ribosome biogenesis and nucleolar hypertrophy in KRAS mutant NSCLC. **a** *Sod1* knockout reduces levels of cytoplasmic 60S subunits and polyribosomes, but not 40S ribosomal subunits. *Sod1*^+/+^ and *Sod1*^*F/F*^ KP cells were treated with 4OHT, their cytoplasmic extracts were prepared 6 days after treatment, and fractionated by a sucrose gradient. **b** *Sod1*^+/+^ and *Sod1*^−/−^ mouse KP lung tumor tissues were analyzed by AgNor staining. Scale bars represent 20 μm. Arrows indicate nucleoli. **c** Quantification of nucleolar size visualized by AgNor staining in *Sod1*^+/+^ and *Sod1*^−/−^ mouse KP lung tumor tissues. Data are shown as mean ± SD; ****p < 0.0001. **d** SOD1 is overexpressed in human primary lung adenocarcinoma (LUCA). SOD1 mRNA expression was compared from 515 human primary LUCA tumors and 59 normal human lung tissues available at the TCGA database. The significance of differences was calculated using two-tailed unpaired Student’s *t* test. **e**–**j** SOD1 expression is positively correlated with that of ribosomal biogenesis genes *RPL26L1*, *NHP2*, *MRTO5*, *POP5*, *EBNA1BP2*, and *RRP1* in the TCGA human LUCA dataset. *n* = 515. Statistical significance was tested using Pearson’s correlation coefficient. The correlation score was analyzed using Pearson’s correlation test. **k** SOD1 expression is positively correlated with the expression of the cell proliferation marker gene *PCNA* in the TCGA human LUCA dataset. *n* = 515. Statistical significance was tested using Pearson’s correlation coefficient. The correlation score was analyzed using Pearson’s correlation test. **l** High SOD1 expression LUCA tumors are associated with poor survival compared with low SOD1 expression in the TCGA human LUCA dataset. Kaplan–Meier analysis was used to compare the overall survival in patients with high (*n* = 239) and low (*n* = 239) SOD1 expression. The *p* value was calculated using the two-sided log-rank test.

Cell growth and proliferation is sensitive to changes in ribosome availability resulting from defects in ribosome biogenesis^38,39^. To assess the impact of SOD1 on the ribosome content of NSCLC cells, we performed sucrose gradient fractionations of cytoplasmic extracts. In mouse *Sod1*^+/+^ KP NSCLC cells, 4OHT treatment did not affect 40S and 60S ribosomal subunits, 80S monoribosomes, or polysomes (Fig. 7a). In contrast, 4OHT effectively reduced the abundance of 60S subunits in *Sod1*^*Flox/Flox*^ KP NSCLC cells, with a concurrent decrease in 80S ribosomes and polysomes (Fig. 7a). This result indicates that *Sod1* knockout selectively impaired biogenesis of 60S ribosomes, which is consistent with SOD1 regulation of PeBoW-dependent pre-rRNA processing to produce 5.8S and 28S mature rRNAs, components of the 60S ribosome subunit. Cancer cells engage in hyperactive ribosome biogenesis to support oncogenic growth, which is characterized by abnormally large nucleoli, known as nucleolar hypertrophy^40^. Nucleolar hypertrophy is a hallmark of human cancer that is associated with poor prognosis^40^. Argyrophilic nucleolar organizer region (AgNOR) staining of nucleoli in tumor tissues is used as a prognostic marker in clinical pathology^41^. In contrast to the large nucleoli in *Sod1*^+/+^ KP lung tumor tissues, nucleoli in *Sod1*^−/−^ tumors were much smaller and more fragmented (Fig. 7b, c), indicating that SOD1 is required for nucleolar hypertrophy and hyperactive ribosome biogenesis in the NSCLC tumors.

To further evaluate the clinical significance of our findings, we examined SOD1 expression and its relationship with ribosome biogenesis in a human LUCA transcriptome dataset from The Cancer Genome Atlas (TCGA). In these lung tumors, SOD1 expression was elevated compared with normal lung tissues (Fig. 7d), and SOD1 expression was positively correlated with the expression of markers for ribosomal biogenesis including RPL26L1, NHP2, MRTO5, POP5, EBNA1BP2, and RRP1 (Fig. 7e–j). In agreement with SOD1 as a key factor for lung cancer cell proliferation, SOD1 level was also positively correlated with that of PCNA, a proliferation marker (Fig. 7k), Moreover, NSCLC tumors with high SOD1 expression correlated with poor survival (Fig. 7l). These observations indicate that SOD1 is important for ribosome biogenesis and proliferation, and is a prognostic marker in human NSCLC tumors.

## Discussion

Growing evidence suggests that SOD1 plays important role in cancer, particularly KRAS-driven NSCLC. SOD1 is overexpressed in lung tumors^7^. Pharmacological inhibition of SOD1 attenuates the growth of NSCLC cells in vitro and lung tumors in mouse models, including those driven by oncogenic KRAS and EGFR^7,10^. This study further provides genetic evidence that SOD1 is critical for KRAS mutant NSCLC tumor development and maintenance. Oncogenic KRAS is known to promote elevated ROS levels in cancer cells due to aberrant metabolism. Activation of antioxidant pathways is generally regarded as a key event during tumorigenesis because tumor cells need to resist excessive ROS and to avoid severe oxidative damage. As the major SOD, the role of SOD1 in cancer has been previously linked to its antioxidant function, and inhibition of SOD1 was shown to increase cancer cell death^2,7–9^. Surprisingly, loss of SOD1 does not significantly affect the level of ROS, and a wide range of commonly used antioxidants could not suppress the growth defect in *Sod1* knockout KP NSCLC cells. Instead, SOD1 is crucial for sustaining the growth of lung cancer cells in KRAS-driven lung tumors in a GEM model as well as cultured human and mouse lung cancer cells. These observations revealed a role of SOD1 in the growth regulation of KRAS-driven NSCLC cells.

SOD1 is known as a classical cytosolic enzyme. Recent studies, however, showed that it is also localized in the nucleus^2^. We found that SOD1 is prominently localized in the nucleus and nucleolus of NSCLC cells, where it interacts with the PeBoW complex that is essential for the processing of pre-rRNA to generate 28S and 5.8S mature rRNAs of the 60S ribosomal subunit. Moreover, SOD1 is required for the recruitment and assembly of the PeBoW complex in the nucleolus. Interestingly, the PeBoW protein complex is involved in coordinating ribosome biogenesis and cell cycle progression^42^. Disrupting the function of this complex by BOP1 and PES1 mutants impairs pre-rRNA processing, biogenesis of 60S ribosomes, and cell proliferation^33,42,43^. Conversely, overexpression of PeBoW components promotes cell proliferation in hepatocellular carcinoma^44^. The nuclear, but not cytoplasmic, form of SOD1 is crucial for the growth function of SOD1 in the KRAS-driven NSCLC. Moreover, the nucleolar form of SOD1 is sufficient to suppress the growth and pre-rRNA processing defects. Consistently, *Sod1* knockout impairs the pre-rRNA processing steps during maturation of 28S and 5.8S rRNAs that require PeBoW function^35,36^, and biogenesis of 60S ribosome subunit, but not 40S ribosome subunit. Together, these observations indicate that PeBoW complex is a key effector for SOD1 to control oncogenic KRAS-driven lung cancer cell proliferation through pre-rRNA processing and 60S ribosome biogenesis.

Ribosomes are biomolecular machines for protein synthesis in all living cells. Mammalian ribosomes are composed of four different rRNAs and ~80 distinct ribosomal proteins (RPs). Ribosome biogenesis starts in the nucleolus where 47S pre-rRNA is transcribed^34^. 47S pre-rRNA is processed to mature rRNAs and assembled with RPs to generate pre-60S and pre-40S ribosomal subunits that are exported to the cytoplasm where they become translationally competent ribosomes^34^. Ribosome biogenesis is one of the most energy-demanding processes in the cell, accounting for up to 90% of nuclear transcriptional activity^45^. Increased nucleolar size or nucleolar hypertrophy is an indicator of hyperactive ribosome biogenesis and a hallmark of cancer that is associated with poor prognosis^46^. *Sod1* ablation impairs ribosome biogenesis and reduces the nucleolar size of KRAS lung tumors. In human LUCA tumors, SOD1 expression is positively correlated with ribosome biogenesis. Collectively, the present study indicates that KRAS-driven lung cancer cells are critically dependent on SOD1 to maintain nucleolar hypertrophy and proliferative capacity.

## Methods

### Mice

All animal care and treatments were carried out in compliance with Rutgers University Institutional Animal Care and Use Committee guidelines. All mice are of C57/B6 and 129 mixed background^47^. UBC-Cre-ERT2 mice^20^ (JAX stock #007001) and *Sod1*^*Flox/Flox*^ mice (kindly provided by Dr. Holly Van Remmen)^19^ were crossbred to generate *UBC-Cre-ERT2*^−/+^ *Sod1*^*Flox/Flox*^ mice. *UBC-Cre-ERT2*^−/+^ *Sod1*^*Flox/Flox*^ mice were further bred with *Kras*^*FSF-G12D/+*^ mice (JAX stock #008653) and *Trp53*^*frt/frt*^ mice (JAX stock #017767) to generate *UBC-Cre-ERT2*^−/+^ *Sod1*^+/+^ *Kras*^*FSF-G12D*/+^ *Trp53*^*frt/frt*^ mice and *UBC-Cre-ERT2*^−/+^ *Sod1*^*Flox/Flox*^ *Kras*^*FSF-G12D*/+^ *Trp53*^*frt/frt*^ mice. These strains used in this study were at the age ranging from 6 to 28 weeks. Both males and females were used. All mice were housed in a pathogen-free unit with a 12 h ligh–dark cycle at a temperature range of 70 ± 2 °F, controlled humidity (30–70%), and supplied with 100% fresh, HEPA (high-efficiency particulate air)-filtered air at 10–15 air changes/h.

To genotype animals, fresh or frozen tail tissue (up to 5 mm) snap-frozen in liquid nitrogen was ground in a pre-chilled pestle and mortar. Genomic DNA was extracted from ground tissue using the NucleoSpin Tissue Kit (Macherey-Nagel, Ref. 740952.10) and analyzed by PCR. TAM was used to activate Cre-ERT2 and initiate Cre/loxP recombination. TAM (Sigma, T5648) was dissolved in sunflower seed oil (Sigma, 88921) with 2% ethanol at 20 mg/ml. Mice were given 160 mg/kg TAM by intraperitoneal injection once a day for 5 consecutive days. To activate KRAS^G12D^ and delete *Trp53* in the lung and produce tumors, mice were infected intranasally with recombinant, replication-deficient Ad-FlpO (University of Iowa Adenoviral Core) at 1.2 × 10^8^ p.f.u. while under anesthesia by isoflurane inhalation^48^.

### Immunological reagents

*For immunohistochemistry and IF*: SOD1 (Abcam, ab16831), 1:500, validated for immunohistochemical (IHC) staining by the manufacturer on human placenta tissue; Ki-67 (Thermo Scientific, MA5-14520), 1:200, validated for IHC staining by the manufacturer on high-grade invasive breast cancer tissue; cleaved caspase-3 (Cell Signaling, 9664), 1:100, validated for IHC staining by the manufacturer on mouse embryo; phospho-histone H2A.X (Cell Signaling, 9718), 1:100, validated for IHC staining by the manufacturer on paraffin-embedded ultraviolet (UV)-treated HT-29 cells; WDR12 antibody, A302-651A (Abcam, ab111955), 1:100, used for IF, validated for IF staining by the manufacturer on A431 cells; BOP1 antibody (Santa Cruz Biotechnology, sc-390672), 1:100, used for IF, validated for IF staining by the manufacturer on HeLa cells; Flag rabbit monoclonal antibody (mAb) (Cell Signaling, 14793), 1:800, used for IF, validated for IF staining by the manufacturer on 293T cells transfected with a GFP-DYKDDDDK-Tag; NPM1 monoclonal antibody (FC-61991) (Thermo Fisher, 32-5200), 1:200, used for IF, validated for IF staining by the manufacturer on A549 and HeLa cells; ApopTag® Plus Peroxidase In Situ Apoptosis Kit (EMD Millipore, S7101) (as per the user guide provided by the manufacturer), validated for IHC staining by the manufacturer on paraffin-embedded human lymph node; biotinylated goat anti-rat IgG antibody, mouse adso–rbed (Vector Labs, BA-9401), validated by the manufacturer against rat heavy- and light-chain IgG.

*For WB*: horseradish peroxidase (HRP)-conjugated glyceraldehyde 3-phosphate dehydrogenase antibody (Proteintech, HRP-60004), 1:5000, validated for WB staining by the manufacturer on various tissues (placenta, heart, and brain) and cell lines (HeLa, H293, and 3T3); phospho-p44/42 MAPK (Erk1/2) (Thr202/Tyr204) (D13.14.4E) XP® rabbit mAb (Cell Signaling, 4370T), 1:1000, validated for WB staining by the manufacturer on COS cells, untreated or treated with either U0126 or TPA; phospho-EGF receptor (Tyr1068) (D7A5) XP® rabbit mAb (Cell Signaling, 3777), 1:1000, validated for WB staining by the manufacturer on BxPC-3 cells, untreated or EGF-stimulated; phospho-p38 MAPK (Thr180/Tyr182) (D3F9) XP® rabbit mAb (Cell Signaling, 4511), 1:1000, validated for WB staining by the manufacturer on COS and 293 cells, untreated or UV treated; SOD1 (Santa Cruz Biotechnology, sc-11407), 1:1000, validated for WB staining by the manufacturer on HeLa, Jurkat, Hs68, and DU I45; WDR12 antibody, A302-651A (Bethyl, A302-651A), 1:2000, validated for WB staining by the manufacturer on HeLa, HEK293T, and mouse NIH3T3; BOP1 antibody, A302-149A (Bethyl, A302-149A), 1:2000, validated for WB staining by the manufacturer on HeLa and HEK293T; exportin-1/CRM1 (D6V7N) rabbit mAb (Cell Signaling, 46249), 1:2000, validated for WB staining by the manufacturer on cell lines (HeLa, HEK293T, NIH/3T3, and COS-7); β-actin (13E5) rabbit mAb (Cell Signaling, 4970), 1:5000, validated for WB staining by the manufacturer on cell lines (NIH/3T3, HeLa, PAE, and A431); HRP-conjugated beta-actin antibody (Proteintech, HRP-66009), 1:5000, validated for WB staining by the manufacturer on cell lines (HeLa, H293, and 3T3).

### Histology

Histology was performed essentially as previously described^49–51^. Briefly, mouse tissues were fixed in 10% neutral buffered formalin solution (Sigma, HT501640) overnight. Fixed tissues were transferred to 75% ethanol and then embedded in paraffin. For frozen sections, tissues were fixed in 10% neutral buffered formalin solution overnight, transferred into 15% sucrose until sinking, and then placed in 30% sucrose overnight. Antibodies used for immunohistochemistry were SOD1 (Abcam, ab16831), Ki-67 (Thermo Scientific, MA5-14520), cleaved caspase-3 (Cell Signaling, 9664), phospho-histone H2A.X (Cell Signaling, 9718), and ApopTag® Plus Peroxidase In Situ Apoptosis Kit (EMD Millipore, S7101). To quantify tumor burden, H&E-stained lung tissue sections were scanned at ×20. Tumor burden was defined as the ratio of tumor area over total lung area. The tumor area and total lung area were outlined and measured using Visiopharm. Pathological analysis and tumor burden quantification were assisted by Biospecimen Repository and Histopathology Services and Biomedical Informatics Shared resources at Rutgers Cancer Institute of New Jersey.

### Nucleolar organizer region (NOR) silver staining

Silver staining of NOR was performed essentially as described^52,53^. Briefly, tissue slides were stained for 10 min at 37 °C with a solution of one volume of 2% gelatin in 1% aqueous formic acid and two volumes of 50% silver nitrate. The slides were then washed several times in ultrapure water, incubated for 10 min in a 5% thiosulfate solution, washed again several times with ultrapure water, and dehydrated and mounted. Slides were then scanned at ×40, and the images were quantified for NOR sizes by ImageJ.

### Tissue protein extraction

Tissues were snap-frozen in liquid nitrogen and lysed in Lysis buffer (50 mM HEPES, 150 mM NaCl, 2 mM EDTA, 1% Triton X-100) with PhosSTOP (Sigma, 4906845001) and cOmplete™ Protease Inhibitor Cocktail (Sigma, 11697498001).

### RNA extraction and RT-qPCR

RNA extraction was performed using the RNeasy Mini Kit (Qiagen, Cat# 74104), following the manufacturer’s instructions. Reverse transcription of messenger RNA (mRNA) used the High-Capacity cDNA Reverse Transcription Kit (Thermo Fisher, Cat# 4368813). The SYBR Green method was used for mRNA quantification. PCR reactions used PowerUp™ SYBR™ Green Master Mix (Thermo Fisher, Cat# A25742) with oligonucleotide primers shown in Supplementary Table 1.

### Mouse and human lung cancer cell lines

Mouse lung cancer cell lines were derived from SOD1fl/fl KP lung tumors in mice. Mouse lung cancer cell lines (KP71-7 and KP71-8) were derived from KP lung tumors in mice. Mouse lung cancer cell lines (KL3-2 and KP3-2) were derived from KL lung tumors in mice. Mouse lung cancer cells were prepared as follows: lung tumors were dissected, and individual tumors were minced into single-cell suspension. Erythrocytes were removed by RBC lysis buffer (155 mM NH_4_Cl, 12 mM NaHCO_3_, 0.1 mM EDTA). Cells were washed with PBS and then placed in culture in RPMI-1640 medium (Thermo Fisher Scientific, Cat# 72400) supplemented with 10% fetal bovine serum and 1% penicillin–streptomycin solution (Thermo Fisher Scientific, Cat# 15140). Human lung cancer cell lines (Calu-1, H1155, A549, CCL-185; H460, H2030, Beas2B, and WI38) were purchased from American Type Culture Collection.

### 4OHT treatment

4OHT (Thermo Fisher Scientific, H7904) was dissolved in tetrahydrofuran (Sigma, 360589) at a concentration of 10 mM, and then diluted to a final concentration of 75 nM with the medium. Tetrahydrofuran was diluted to the same extent to serve as a vehicle control. Following overnight 4OHT incubation, cells were either washed with PBS then replenished with fresh medium or were trypsinized and reseeded.

### Cell lysis

Cell were lysed with a lysis buffer comprised of 20 mM Tris-HCl (pH 7.5), 150 mM NaCl, 1 mM Na_2_EDTA, 1 mM EGTA, 1% NP-40, 1% sodium deoxycholate, and 1 mM PMSF. PhosSTOP (Sigma, 4906845001) and cOmplete™ Protease Inhibitor Cocktail (Sigma, 11697498001) were added, according to the application notes.

### Cell growth assay

Cell growth was measured by a sulforhodamine B (SRB) colorimetric assay^54^. Cells were seeded in triplicate at a predetermined density (500–2000 cells per well) in 96-well plates. Cells were fixed in place with 100 μl 10% trichloroacetic acid (Sigma, T6399) at 4 °C for 1 h. After washing and air drying, cells were then stained with 50 μl of 0.057% (wt/vol) SRB (Sigma, S1402, dissolved in 1% acetic acid) for 30 min. Unbound dye was washed away once with water and three times in 1% acetic acid. The cells were allowed to air dry before the dye was solubilized by adding 200 μl of 10 mM Tris base solution (pH 10.5). Conversion of colored end product was measured by optical density at 510 nm in a TECAN Infinite M200PRO plate reader.

### Crystal violet colony staining

Cells adherent to 96- or 12-well plates were washed once with PBS, and then stained with 0.5% crystal violet staining solution in 20% methanol for 20 min with agitation. Unbound dye was washed away ten times with water, and cells were allowed to air dry.

### ROS measurement

Intracellular superoxide was measured using DHE (Thermo Fisher Scientific, D1168). Cells (1 × 10^5^/well) were seeded into black-walled clear-bottom 96-well plates (Costar, 3603). Cells were incubated in RPMI-1640 medium without phenol red or serum, with 5 mM DHE for 10 min before reading on a TECAN infinite M200PRO plate reader at excitation and emission wavelengths of 500/580 nm, respectively. Total ROS was determined using CM-H_2_DCFDA (Thermo Fisher Scientific, C6827). Cells (1 × 10^5^/well) were seeded into black-walled clear-bottom 96-well plates (Costar, 3603). Cells were incubated in RPMI-1640 medium without phenol red or serum, with 5 mM CMH2-DCFDA for 45 min before reading at excitation and emission wavelengths of 495/529 nm, respectively. Fluorescence readings were normalized to total cell number, as measured by the SRB assay^54^. The data were collected and analyzed by Infinite reader i-control software ver.1.12 (Tecan).

### Plasmids

Human SOD1 was cloned into the lentiviral vector pInducer20 (Addgene, Cat# 44012). SOD1 tag was generated by PCR, and mutations by site-directed mutagenesis using QuikChange (Agilent Technologies) with oligonucleotide primers shown in Supplementary Fig. 1. NoLS-Sod1 was constructed using NEBuilder® HiFi DNA Assembly (NEB) from the PCR-amplified pSBtet-GP expression cassette (Addgene, Cat# 60520), mouse Sod1 coding sequence, and oligonucleotides encoding an N-terminal NoLS MPKKKRKVPHRRRRRRR and a C-terminal Flag tag. Next, the puromycin marker in the pSBtet vector was replaced with a blasticidin resistance marker amplified from pCDNA6/TR (Thermo Fisher Scientific) via the NEBuilder® protocol.

### Establishment of stable Tet-on cells

To generate lentiviruses, SOD1 plasmid DNA was transfected along with packaging plasmids psPAX2 (Addgene, Cat# 12260), and pMD2.G (Addgene, Cat# 12259) into HEK293T cells using Fugene 6 (Promega, Cat# E2691). Viral supernatant was collected at 48 and 72 h after transfection and mouse lung cancer cells were transiently infected twice in the presence of 8 μg/ml polybrene (Sigma). Transfected cells were selected with 50 μg/ml G418 (Thermo Fisher Scientific, Cat# 10131035) at 48 h after viral infection for 2 weeks. To generate stable Dox-inducible NoLS-Sod1 cells, mouse lung cancer cells were cotransfected with pSBtet-NoLS-Sod1-Flag and the Sleeping Beauty transposase plasmid pCMV(CAT)T7-SB100 (Addgene, Cat# 34879) using PolyFect (Qiagen), followed by selection with 10 µg/ml blasticidin for 7 days.

### Anti-Flag-SOD1 immunoprecipitation and mass spectrometry analysis

Cells expressing TET-inducible Flag-SOD1 were treated with 4OHT overnight. The fresh medium with 500 ng/ml Dox was used to induce expression of Flag-SOD1. Untreated cells were used to serve as the control. After 5 days, cells were collected 2 h after replacement of fresh medium. Cells were lysed in 300 μl of ice-cold Buffer A (40 mM HEPES [pH 7.5], 120 mM NaCl, 1 mM EDTA, 1% Triton X-100, one tablet PhosSTOP^TM^ (Roche) per 10 ml and one tablet EDTA-free protease inhibitors (Roche) per 10 ml) with 2.5 mg/ml DSP, and incubated for 1 h on ice. The crosslinking reaction was quenched by adding 75 μl 1 M Tris-HCl (pH 7.4) for 30 min. After centrifugation to clear-cell debris, 40 μl of ANTI-FLAG® M2 Affinity Gel (Sigma, 2220) was added to the supernatant and incubated with rotation overnight at 4 °C. Immunoprecipitates were washed once each with Tris-buffered saline containing 0.05% Tween-20, Wash Buffer 1 (50 mM HEPES [pH 7.5], 40 mM NaCl, and 2 mM EDTA) with 1% Triton X-100, Wash Buffer 1 with 500 mM LiCl and 0.5% Triton X-100, Wash Buffer 1 with 500 mM LiCl, and Wash Buffer 2 (50 mM HEPES [pH 7.5] and 150 mM NaCl). Flag beads were eluted with 0.1 M glycine (pH 2.5) and neutralized with 0.1 volume of 1 M Tris-HCl (pH 8.5). Elution was analyzed by LC-MS/MS at the Biological Mass Spectrometry facility of Rutgers Robert Wood Johnson Medical School.

### Analysis of ribosome biogenesis

Northern blotting was performed as previously described^37^. The hybridization signals were analyzed by phosphorimaging using a Typhoon biomolecular imager and ImageQuant software ver. 2.0.0.6 (Cytiva)(GE Healthcare), ImageQuant TL ver. 8.2 (Cytiva) and ImageQuant ver. 5.2 (Molecular Dynamics). Image processing was performed with Canvas ver. X.898 (ACD Systems, Inc.) . For oligonucleotide probe sequences, see Supplementary Table 1. Cytoplasmic ribosomes were analyzed using an established procedure^35^ with a few modifications as follows. Cycloheximide (CHX) was added to the culture medium to 50 μg/ml immediately prior to cell harvesting, after which cells were trypsinized and pelleted in ice-cold RPMI containing 50 μg/ml CHX at 1500 × *g* for 4 min. Cells were resuspended in 1 ml ice-cold buffer A1 (20 mM Tris-HCl pH 7.4, 130 mM KCl, 10 mM MgCl_2_, 50 μg/ml CHX), transferred to a microcentrifuge tube, and pelleted at 1150 × *g* for 2 min at 4 °C. Cells were then lysed in 200 μl buffer A1 containing 0.5% Igepal CA-630, 0.5% sodium deoxycholate, 2.5 mM dithiothreitol (DTT), 0.2 mg/ml heparin, and 80 U/ml RiboLock (Thermo Fisher) for 10 min with nutation at 4 °C. Cell lysate was cleared by centrifugation at 10,000 × *g* for 10 min at 4 °C. The supernatant was layered on top of a 15–50% (w/v) sucrose gradient in 10 mM Tris-HCl pH 7.4, 60 mM KCl, 10 mM MgCl_2_, 1 mM DTT, 0.2 mg/ml heparin, and 0.01% Brij 35. The gradients were centrifuged at 160,000 × *g* for 200 min at 4 °C in a Beckman SW41Ti rotor. The gradients were fractionated by upward displacement with 69% (w/v) sucrose in a Beckman Fraction Recovery System. The *A*_254_ was measured using a BioRad EM-1 UV cell and the data were analyzed with WinDaq ver. 3.76 (DATAQ Instruments, Inc.).

### IF and Duolink proximal ligation assay (PLA)

IF was performed as previously described^55–58^. PLA was based on a published protocol^32^. Briefly, KP NSCLC cells stably transfected with Pinducer20-Flag-SOD1 were treated with 4OHT overnight. Cells were then trypsinized and seeded on coverslips, and Dox was added to the medium to induce Flag-SOD1 expression. Cells were fixed using 1% paraformaldehyde after growth for 5 days. Cells were blocked with 10% goat serum in PBS with 0.1% Triton X-100. The coverslips were incubated in primary antibodies as indicated overnight at 4 °C. Antibodies used were anti-Flag (mouse F1804, Sigma-Aldrich, USA and rabbit PA1-984B, Thermo Fisher), anti-WDR12 (A302-651A, Bethyl Laboratories), anti-BOP1 (A302-149, Bethyl Laboratories), and anti-NPM1 (FC-61991, Thermo Fisher). Cells not treated with 4OHT or Dox were used as a negative control. Duolink® In Situ Red Starter Kit Mouse/Rabbit (DUO92101, Sigma-Aldrich, USA) was used for amplification and detection of bound PLA (proximity ligation amplification) probes. Confocal images were captured by Nikon A1R-Si Confocal Microscope System.

### Expression analysis of SOD1 and ribosome biogenesis in human primary LUCA datasets

SOD1 mRNA expression was compared with mRNAs of ribosome biogenesis or PCNA from 515 human primary LUCA tumors and 59 normal human lung tissues available at TCGA database [https://xenabrowser.net/datapages/?dataset=TCGA.LUAD.sampleMap%2FHiSeqV2&host=https%3A%2F%2Ftcga.xenahubs.net&removeHub=https%3A%2F%2Fxena.treehouse.gi.ucsc.edu%3A443]. High SOD1 expression LUCA tumors are associated with poor survival compared with low SOD1 expression in the TCGA human LUCA dataset. Kaplan–Meier analysis was used to compare overall survival in patients with high (*n* = 239) and low (*n* = 239) SOD1 expression was investigated using the following GEPIA human LUCA dataset [http://gepia.cancer-pku.cn/detail.php?gene=&clicktag=survival].

### Statistical analysis

Statistical analyses were carried out using the GraphPad Prism 8.43 software. Unless otherwise indicated, differences between treatment groups were compared using two-tailed Student’s *t* test.

### Reporting summary

Further information on research design is available in the Nature Research Reporting Summary linked to this article.

## Supplementary information

Supplementary Information

Reporting Summary

## Data Availability

The mass spectrometry data have been deposited in the MassIVE database under the accession identifier MSV000087044. The mRNA expression data of human primary LUCA tumors referenced during the study are available in the public repository TCGA database [https://xenabrowser.net/datapages/?dataset=TCGA.LUAD.sampleMap%2FHiSeqV2&host=https%3A%2F%2Ftcga.xenahubs.net&removeHub=https%3A%2F%2Fxena.treehouse.gi.ucsc.edu%3A443]. Data supporting the findings of this study are available within the article and its Supplementary information files and from the corresponding author upon reasonable request. A Reporting summary for this article is available as a Supplementary information file. Source data are provided with this paper.

## Source data

Source Data

## References

[1] Crapo JD, Oury T, Rabouille C, Slot JW, Chang LY (1992). Copper, zinc superoxide dismutase is primarily a cytosolic protein in human cells. Proc. Natl Acad. Sci. USA.

[2] Tsang CK, Liu Y, Thomas J, Zhang Y, Zheng XFS (2014). Superoxide dismutase 1 acts as a nuclear transcription factor to regulate oxidative stress resistance. Nat. Commun..

[3] Miao L, St. Clair DK (2009). Regulation of superoxide dismutase genes: Implications in disease. Free Radic. Biol. Med..

[4] Che M, Wang R, Li X, Wang HY, Zheng XF (2016). Expanding roles of superoxide dismutases in cell regulation and cancer. Drug Discov. Today.

[5] Juarez JC (2008). Superoxide dismutase 1 (SOD1) is essential for H2O2-mediated oxidation and inactivation of phosphatases in growth factor signaling. Proc. Natl Acad. Sci. USA.

[6] Reddi AR, Culotta VC (2013). SOD1 integrates signals from oxygen and glucose to repress respiration. Cell.

[7] Glasauer A, Sena LA, Diebold LP, Mazar AP, Chandel NS (2014). Targeting SOD1 reduces experimental non-small-cell lung cancer. J. Clin. Invest..

[8] Gomez ML, Shah N, Kenny TC, Jenkins EC, Germain D (2019). SOD1 is essential for oncogene-driven mammary tumor formation but dispensable for normal development and proliferation. Oncogene.

[9] Papa L, Hahn M, Marsh EL, Evans BS, Germain D (2014). SOD2 to SOD1 switch in breast cancer. J. Biol. Chem..

[10] Somwar R (2011). Superoxide dismutase 1 (SOD1) is a target for a small molecule identified in a screen for inhibitors of the growth of lung adenocarcinoma cell lines. Proc. Natl Acad. Sci. USA.

[11] Tsang CK (2018). SOD1 phosphorylation by mTORC1 couples nutrient sensing and redox regulation. Mol. Cell.

[12] Bray, F. et al. Global cancer statistics 2018: GLOBOCAN estimates of incidence and mortality worldwide for 36 cancers in 185 countries. *CA Cancer J*. *Clin*. **0**, 1–31 (2018). 10.3322/caac.21492 30207593

[13] Gridelli C (2015). Non-small-cell lung cancer. Nat. Rev. Dis. Prim..

[14] The Cancer Genome Atlas Research, N. (2014). Comprehensive molecular profiling of lung adenocarcinoma. Nature.

[15] Juarez JC (2006). Copper binding by tetrathiomolybdate attenuates angiogenesis and tumor cell proliferation through the inhibition of superoxide dismutase 1. Clin. Cancer Res..

[16] Huang P, Feng L, Oldham EA, Keating MJ, Plunkett W (2000). Superoxide dismutase as a target for the selective killing of cancer cells. Nature.

[17] Lee K (2013). The copper chelator ATN-224 induces peroxynitrite-dependent cell death in hematological malignancies. Free Radic. Biol. Med..

[18] Karsli-Uzunbas G (2014). Autophagy is required for glucose homeostasis and lung tumor maintenance. Cancer Discov..

[19] Zhang Y (2013). CuZnSOD gene deletion targeted to skeletal muscle leads to loss of contractile force but does not cause muscle atrophy in adult mice. FASEB J..

[20] Ruzankina Y (2007). Deletion of the developmentally essential gene ATR in adult mice leads to age-related phenotypes and stem cell loss. Cell Stem Cell.

[21] Borchelt DR (1994). Superoxide dismutase 1 with mutations linked to familial amyotrophic lateral sclerosis possesses significant activity. Proc. Natl Acad. Sci. USA.

[22] Rodriguez-Lara V, Hernandez-Martinez J-M, Arrieta O (2018). Influence of estrogen in non-small cell lung cancer and its clinical implications. J. Thorac. Dis..

[23] Itoh K (1999). Keap1 represses nuclear activation of antioxidant responsive elements by Nrf2 through binding to the amino-terminal Neh2 domain. Genes Dev..

[24] Li L (2014). Nrf2/ARE pathway activation, HO-1 and NQO1 induction by polychlorinated biphenyl quinone is associated with reactive oxygen species and PI3K/AKT signaling. Chem. Biol. Interact..

[25] Furukawa Y, T AS, O’Halloran TV (2004). Oxygen-induced maturation of SOD1: a key role for disulfide formation by the copper chaperone CCS. EMBO J..

[26] Valentine JS, Doucette PA, Zittin Potter S (2005). Copper-zinc superoxide dismutase and amyotrophic lateral sclerosis. Annu. Rev. Biochem..

[27] Ratovitski T (1999). Variation in the biochemical/biophysical properties of mutant superoxide dismutase 1 enzymes and the rate of disease progression in familial amyotrophic lateral sclerosis kindreds. Hum. Mol. Genet..

[28] Lapik YR, Fernandes CJ, Lau LF, Pestov DG (2004). Physical and functional interaction between Pes1 and Bop1 in mammalian ribosome biogenesis. Mol. Cell.

[29] Hölzel M (2005). Mammalian WDR12 is a novel member of the Pes1–Bop1 complex and is required for ribosome biogenesis and cell proliferation. J. Cell Biol..

[30] Fan P, Wang B, Meng Z, Zhao J, Jin X (2018). PES1 is transcriptionally regulated by BRD4 and promotes cell proliferation and glycolysis in hepatocellular carcinoma. Int. J. Biochem. Cell Biol..

[31] Killian A (2006). Contribution of the BOP1 gene, located on 8q24, to colorectal tumorigenesis. Genes Chromosomes Cancer.

[32] Gullberg M, Göransson C, Fredriksson S (2011). Duolink-“In-cell Co-IP” for visualization of protein interactions in situ. Nat. Methods.

[33] Rohrmoser M (2007). Interdependence of Pes1, Bop1, and WDR12 controls nucleolar localization and assembly of the PeBoW complex required for maturation of the 60S ribosomal subunit. Mol. Cell. Biol..

[34] Henras AK, Plisson-Chastang C, O’Donohue M-F, Chakraborty A, Gleizes P-E (2015). An overview of pre-ribosomal RNA processing in eukaryotes. Wiley Interdiscip. Rev. RNA.

[35] Strezoska Z, Pestov DG, Lau LF (2000). Bop1 is a mouse WD40 repeat nucleolar protein involved in 28S and 5. 8S RRNA processing and 60S ribosome biogenesis. Mol. Cell. Biol..

[36] Wang M, Anikin L, Pestov DG (2014). Two orthogonal cleavages separate subunit RNAs in mouse ribosome biogenesis. Nucleic Acids Res..

[37] Wang M, Pestov DG (2016). Quantitative Northern blot analysis of mammalian rRNA processing. Methods Mol. Biol..

[38] Baßler J, Hurt E (2019). Eukaryotic ribosome assembly. Annu. Rev. Biochem..

[39] Mills EW, Green R (2017). Ribosomopathies: there’s strength in numbers. Science.

[40] Montanaro L, Treré D, Derenzini M (2008). Nucleolus, ribosomes, and cancer. Am. J. Pathol..

[41] Derenzini M, Montanaro L, Treré D (2009). What the nucleolus says to a tumour pathologist. Histopathology.

[42] Holzel M (2005). Mammalian WDR12 is a novel member of the Pes1-Bop1 complex and is required for ribosome biogenesis and cell proliferation. J. Cell Biol..

[43] Grimm T (2006). Dominant-negative Pes1 mutants inhibit ribosomal RNA processing and cell proliferation via incorporation into the PeBoW-complex. Nucleic Acids Res..

[44] Yin Y, Zhou L, Zhan R, Zhang Q, Li M (2018). Identification of WDR12 as a novel oncogene involved in hepatocellular carcinoma propagation. Cancer Manage. Res..

[45] Pelletier J, Thomas G, Volarević S (2017). Ribosome biogenesis in cancer: new players and therapeutic avenues. Nat. Rev. Cancer.

[46] Pianese, G. *Beitrag zur histologie und aetiologie des carcinoms*, Vol. 1 (Fischer, 1896).

[47] Simpson EM (1997). Genetic variation among 129 substrains and its importance for targeted mutagenesis in mice. Nat. Genet..

[48] DuPage M, Dooley AL, Jacks T (2009). Conditional mouse lung cancer models using adenoviral or lentiviral delivery of Cre recombinase. Nat. Protoc..

[49] Thomas JaniceD (2014). Rab1A is an mTORC1 activator and a colorectal oncogene. Cancer Cell.

[50] Wu T-J (2015). Identification of a non-gatekeeper hot spot for drug-resistant mutations in mTOR kinase. Cell Rep..

[51] Zhang H (2018). Significance and mechanism of androgen receptor overexpression and androgen receptor/mechanistic target of rapamycin cross‐talk in hepatocellular carcinoma. Hepatology.

[52] Derenzini M (1998). Nucleolar function and size in cancer cells. Am. J. Pathol..

[53] Trere D (2000). AgNOR staining and quantification. Micron.

[54] Vichai V, Kirtikara K (2006). Sulforhodamine B colorimetric assay for cytotoxicity screening. Nat. Protoc..

[55] Li H, Tsang C, Watkins M, Bertram P, Zheng X (2006). Nutrient regulates Tor1 nuclear localization and association with rDNA promoter. Nature.

[56] Tsang C, Li H, Zheng X (2007). Nutrient starvation promotes condensin loading to maintain rDNA stability. EMBO J..

[57] Tsang CK, Zheng XFS (2009). Opposing role of condensin and radiation-sensitive gene RAD52 in ribosomal DNA stability regulation. J. Biol. Chem..

[58] Wei Y, Tsang C, Zheng X (2009). Mechanisms of regulation of RNA polymerase III-dependent transcription by TORC1. EMBO J..

