# Supplementary material for: SOD1 regulates ribosome biogenesis in KRAS mutant non-small cell lung cancer
Source: Nat Commun. 2021 Apr 15;12:2259. doi: 10.1038/s41467-021-22480-x (PMC8050259; doi:10.1038/s41467-021-22480-x)
Supplement: Supplementary file 1 — Supplementary Information [file 41467_2021_22480_MOESM1_ESM.pdf]

Supplementary Information

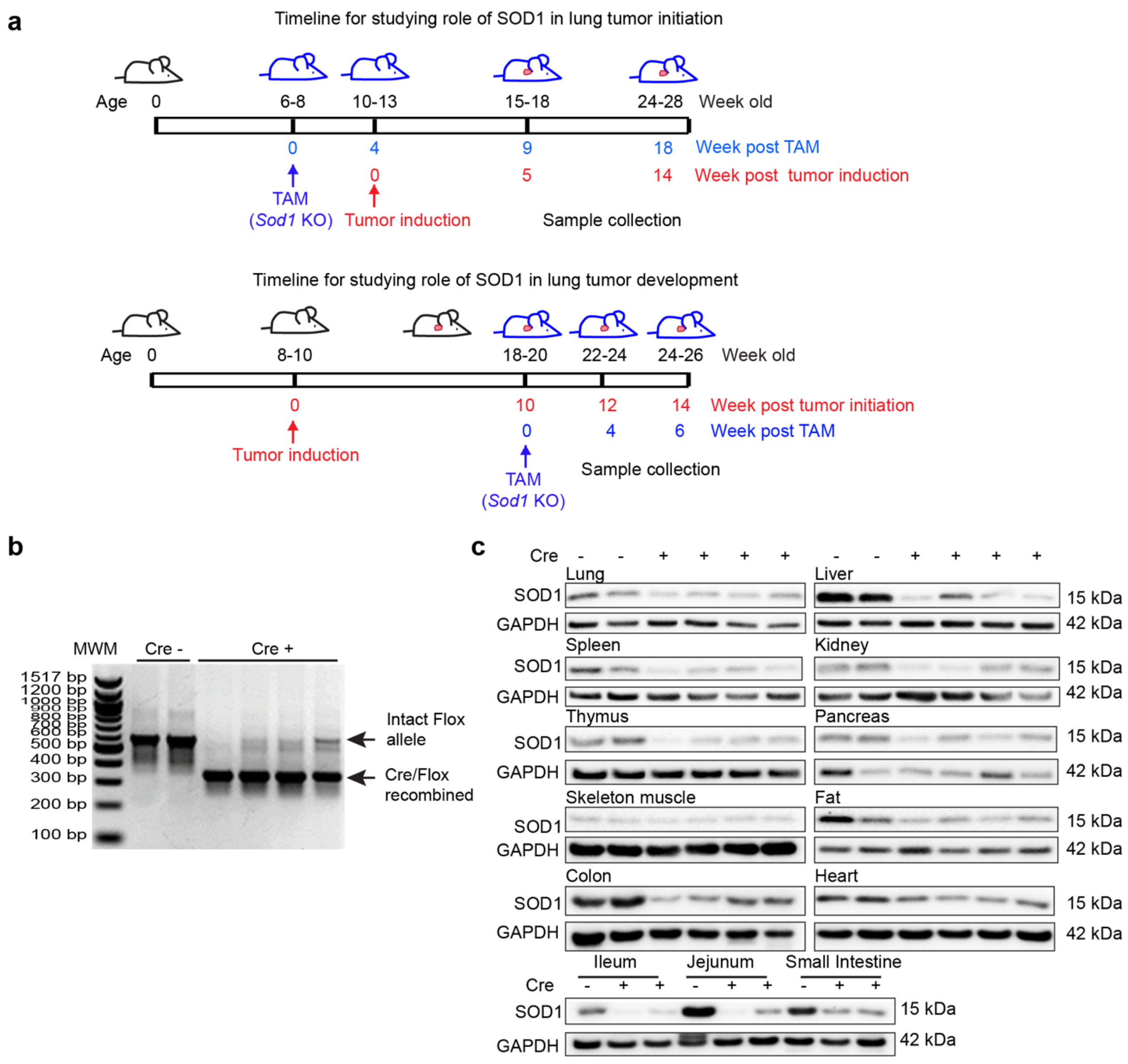

Supplementary Figure 1. UBC-Cre-ERT2-facilitated global *Sod1* knockout in *Sod1*$^{Flox/Flox}$ mice by TAM

a Experimental scheme for examining the effect of *Sod1* knockout on lung tumor initiation (upper panel) and development (lower panel).

b Genotyping PCR analysis of tail genomic DNA. MWM, molecular weight marker. Data represent three independent experiments.

c Western blot analysis of SOD1 protein in various tissues from *Sod1*$^{Flox/Flox}$ mice and *Sod1*$^{Flox/Flox}$ *UBC-Cre-ERT2* mice two weeks post TAM. Data represents Cre^{-}: n =2, Cre^{+}: n = 4.

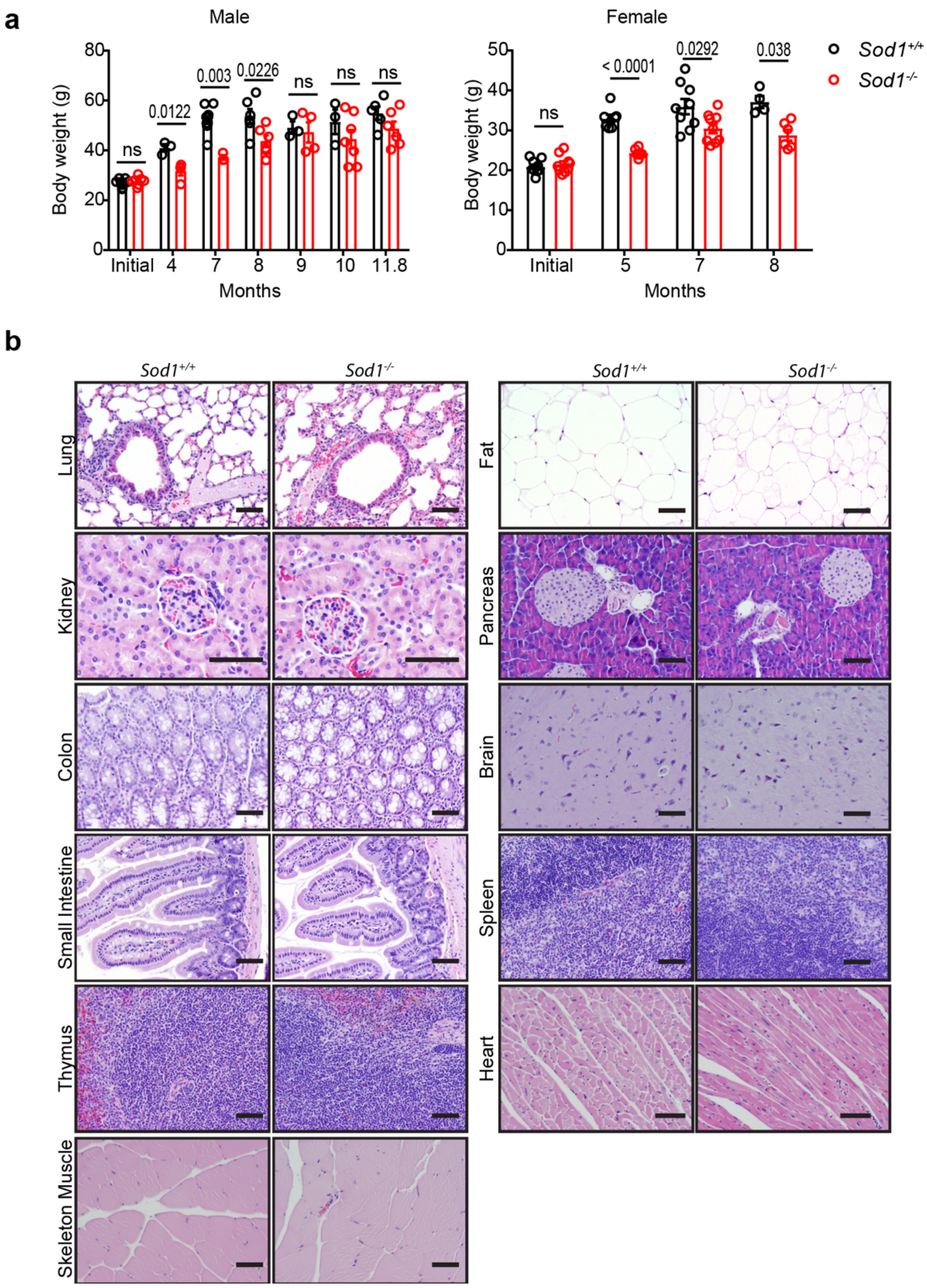

Supplementary Figure 2. No apparent histological defects in major tissues after inducible *Sod1* knockout

a Body weight of *Sod1* knockout mice and control mice was monitored for up to 9 months post tamoxifen injection. Data presented as mean ± SEM; Statistical significance was tested using two-tailed unpaired t test. *$p<0.05$, **$p<0.001$, ***$p<0.0001$. p values for groups with significant difference: male, 4 months: $p = 0.0122$; 7 month, $p = 0.003$; 8 month, $p = 0.0226$; female: 5 month, $p < 0.0001$; 7 month, p = 0 0292; 8 month, $p = 0.038$.

b Representative H&E staining of indicated mouse tissues at 5-months post TAM. Scale bar: 100 μm. n = 3 mice per mouse group. Representative data from each group are shown.

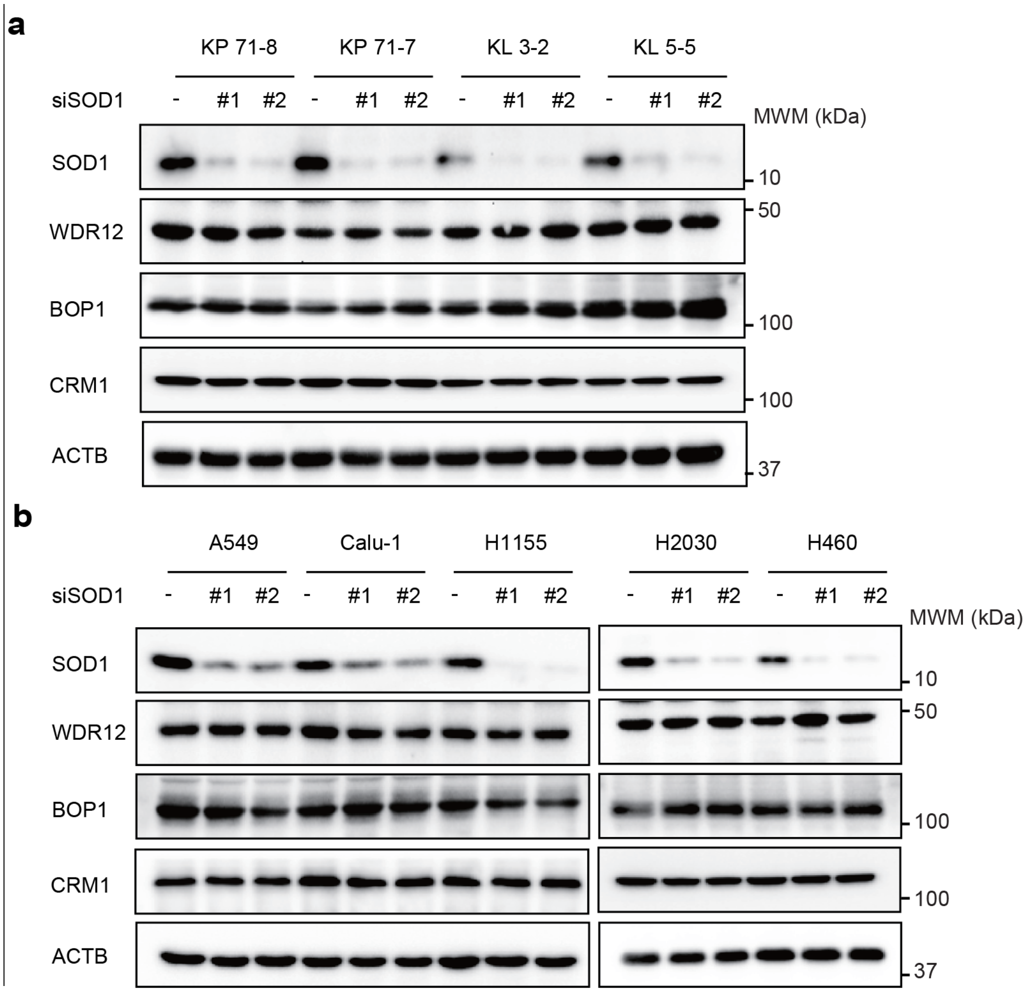

Supplementary Figure 3. Knockdown of SOD1 in mouse and human KP and KL NSCLC cell lines

a Mouse KP and KL NSCLC cells were transfected with a control siRNA or two different SOD1 siRNAs for 6 days. Protein expression of SOD1, WDR12, BOP1 and CRM1 was analyzed by Western blot. ACTB was used as a loading control.

b Human KP and KL NSCLC cells were transfected with a control siRNA or two different SOD1 siRNAs for 6 days. Protein expression of SOD1, WDR12, BOP1 and CRM1 was analyzed by Western blot. ACTB was used as a loading control.

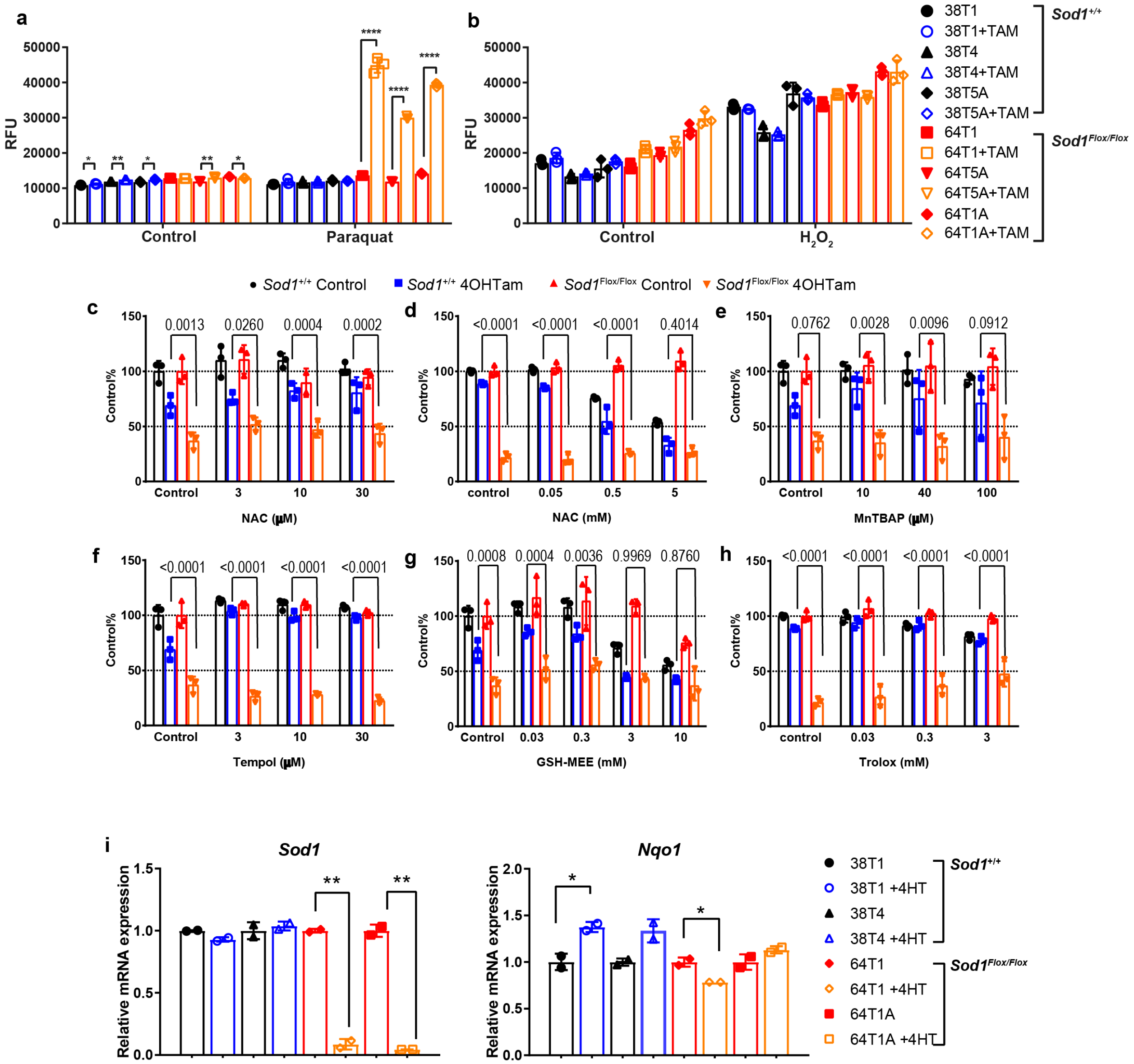

Supplementary Figure 4. *Sod1* knockout does not significantly alter ROS levels but impairs anti-superoxide capacity in KP cells

a Effect of *Sod1* knockout on cellular superoxide level. 6 days after 4OHT treatment, 3 clones each of *Sod1*$^{+/+}$ and *Sod1*$^{Flox/Flox}$ KP NSCLC cells were stained with DHE following treatment with or without 1 mM paraquat for 1 hour. Data represent means ± SD (standard deviation) of three independent experiments. Statistical significance was tested using two-tailed unpaired t test. *p<0.05, **p<0.01, ***p<0.001, ****p<0.0001.

b Effect of*Sod1* knockout on general ROS levels. 6 days after 4OHT treatment, 4 clones each of *Sod1*$^{+/+}$ and *Sod1*$^{Flox/Flox}$ KP NSCLC cells were stained with CM-H_2DCFDA following treatment with or without 0.5 mM H_2O_2 for 15 min. Data represent means ± SD (standard deviation) of three independent experiments.

c-h Antioxidants fail to suppress the growth defect of *Sod1* knockout KP NSCLC cells. 4OHT or vehicle-treated *Sod1*$^{+/+}$ or *Sod1*$^{Flox/Flox}$ KP NSCLC cells were treated for 7 days with different concentrations of the following antioxidants: N-acetylcysteine (NAC) (**c** and **d**), MnTBAP (**e**), Tempol (**f**), GSH-MEE (**g**), or Trolox (**h**). Cell growth was measured by SRB assay. Data represent means ± SD of three independent experiments. Statistical significance was tested using two-tailed unpaired t test. p values are shown to indicate the statistical significance in growth of 4OHT treated control and *Sod1* knockout cells.

i Quantification of *Sod1* and *Nqo1* mRNA levels in 4OHT or vehicle treated *Sod1*$^{+/+}$ or *Sod1*$^{Flox/Flox}$ NSCLC cells. Data shown as mean ± SD of two independent experiments. Statistical significance was tested using two-tailed unpaired t test. * $p<0.05$, ** $p<0.01$, *** $p<0.001$, **** $p<0.0001$.

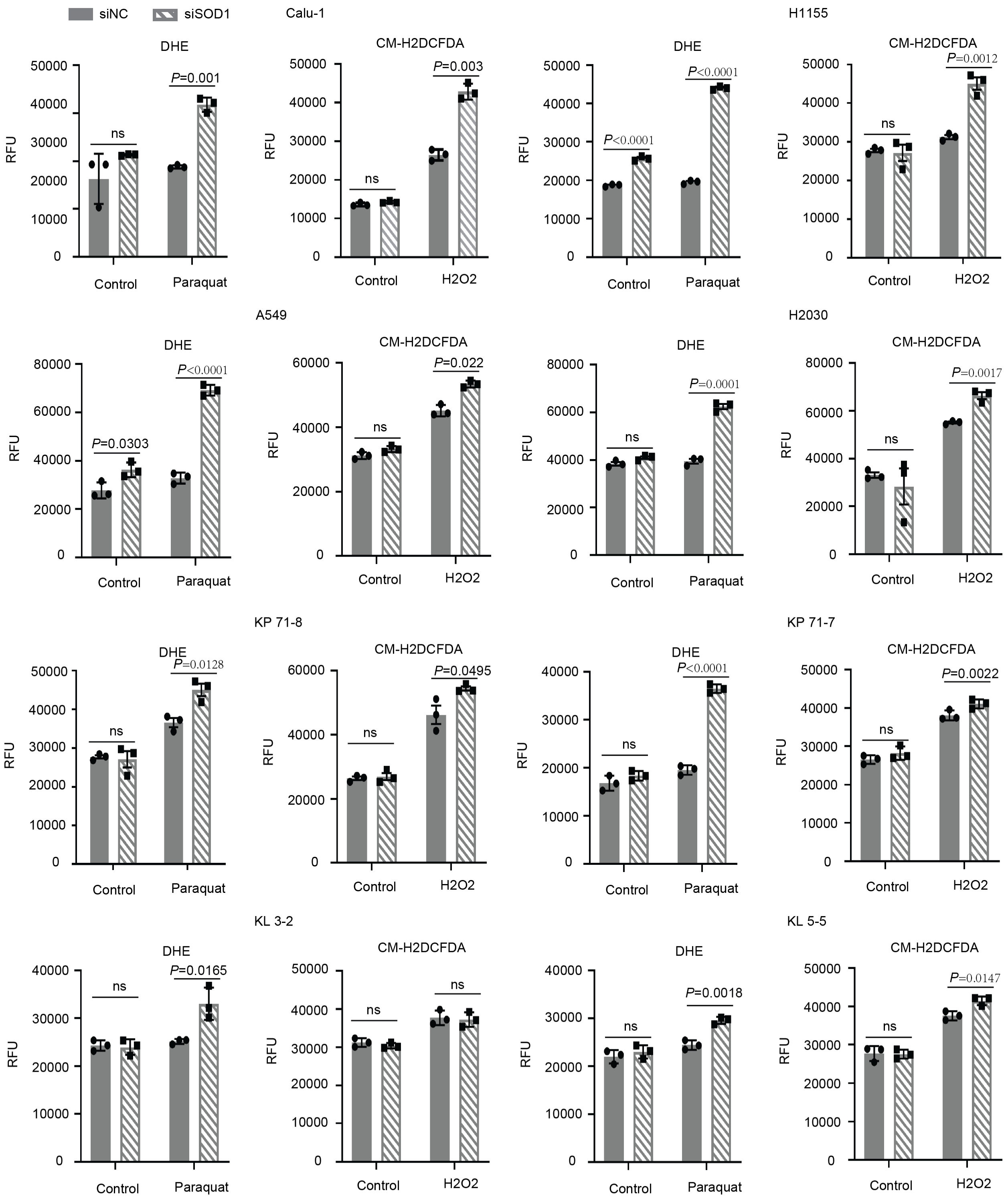

Supplementary Figure 5. SOD1 knockdown does not significantly increase ROS levels but impairs anti-superoxide capacity in human and mouse KP and KL NSCLC cells.

Human and mouse KP and KL NSCLC cell lines were transfected with SOD1 siRNA (siSOD1) or control siRNA (siNC) for 6 days. Cells were treated with or without 1 mM paraquat for 1 hour and stained with the superoxide dye DHE, or treated with or without 0.5 mM H_2O_2 for 15 min and stained with the general ROS dye CM-H_2DCFDA. Data are presented as the mean ± SEM (n = 3) of two independent experiments. The significance of differences was calculated using two-tailed unpaired Student's t-test. ns, not significant.

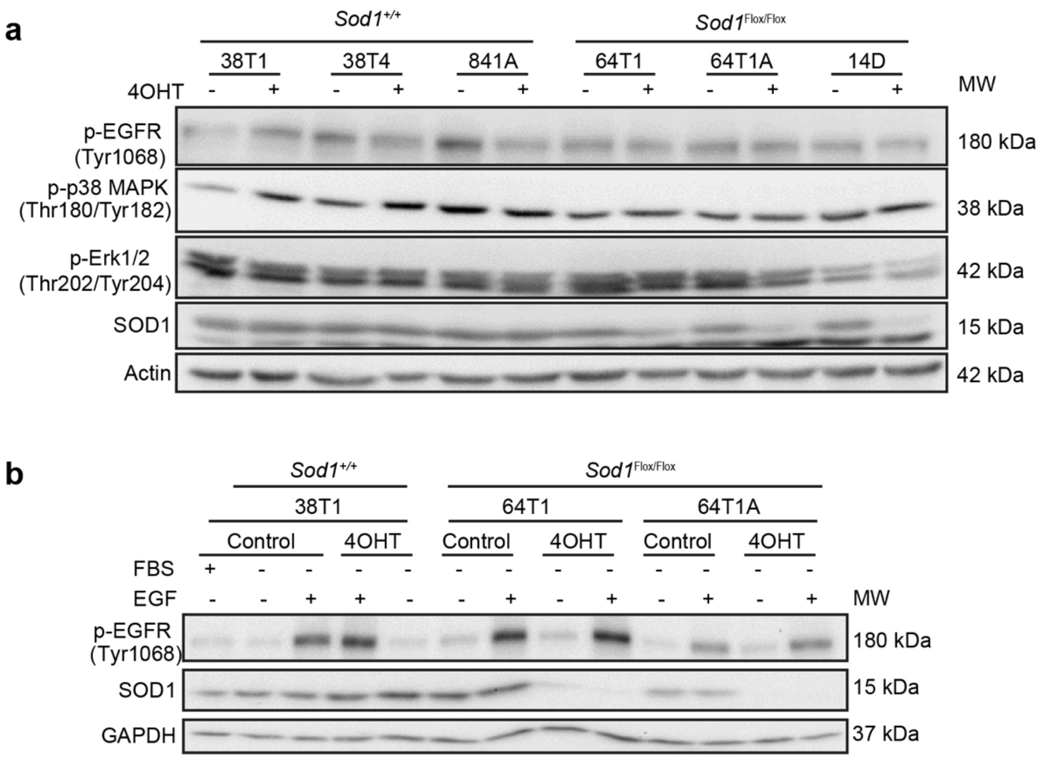

Supplementary Figure 6. SOD1 is not required for phosphorylation of EGFR, ERK and p38MAPK

a Western blot analysis of p-EGFR, p-p38, p-ERK1/2, SOD1 and actin levels in *Sod1*$^{+/+}$ or *Sod1*$^{Flox/Flox}$ KP NSCLC cells treated with 4OHT or vehicle. Data represent three independent experiments.

b Western blot analysis of phospho-EGFR, SOD1 and GAPDH protein levels in *Sod1*$^{+/+}$ and *Sod1*$^{Flox/Flox}$ KP NSCLC cells treated with 4OHT or vehicle and cultured in full medium (containing fetal bovine serum, FBS), FBS starvation conditions or with EGF stimulation (100ng/ml for 10 min). Data represent three independent experiments.

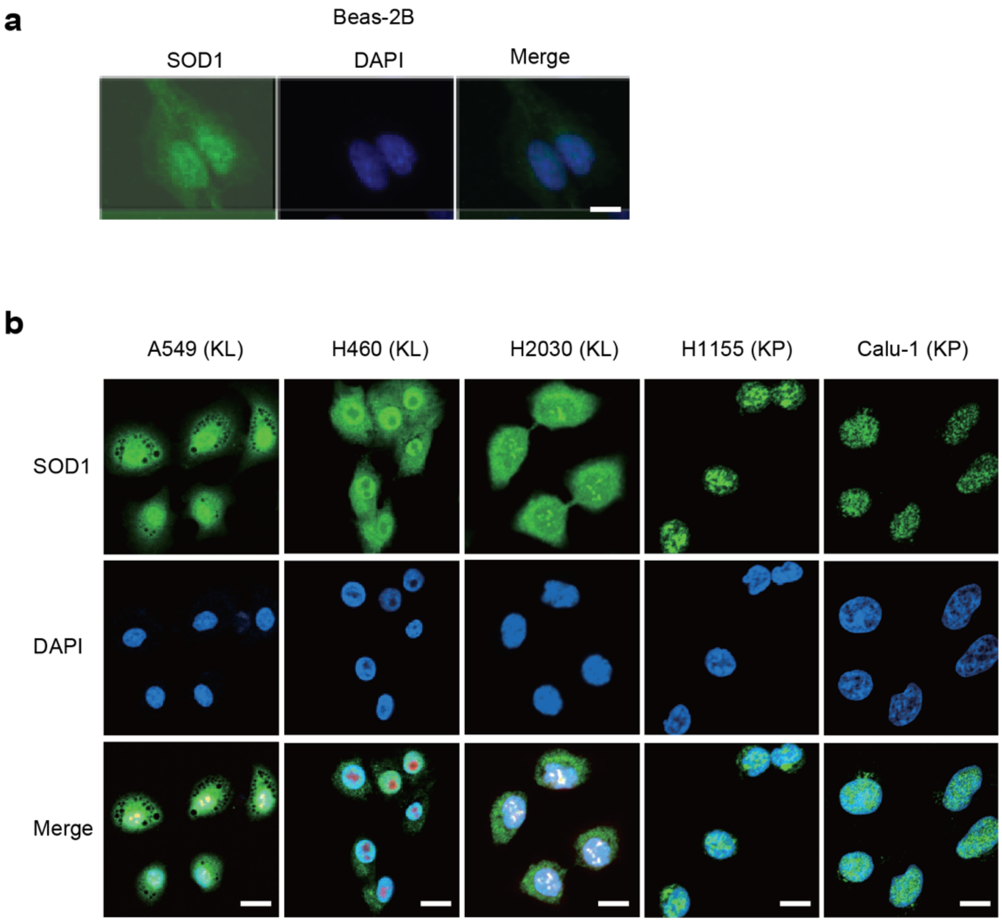

Supplementary Figure 7. SOD1 localization in human immortalized airway lung cells and KL and KP NSCLC cells

Beas-2B (human immortalized airway lung cells) cells (**a**), and A549 (KL), H460 (KL), H2030 (KL), H1155 (KP) and Calu-1 (KP) NSCLC cells (**b**) were analyzed by IF staining with SOD1 antibody. Nuclei were stained with DAPI. Scale bar represents 10 µm. Data represent two independent experiments.

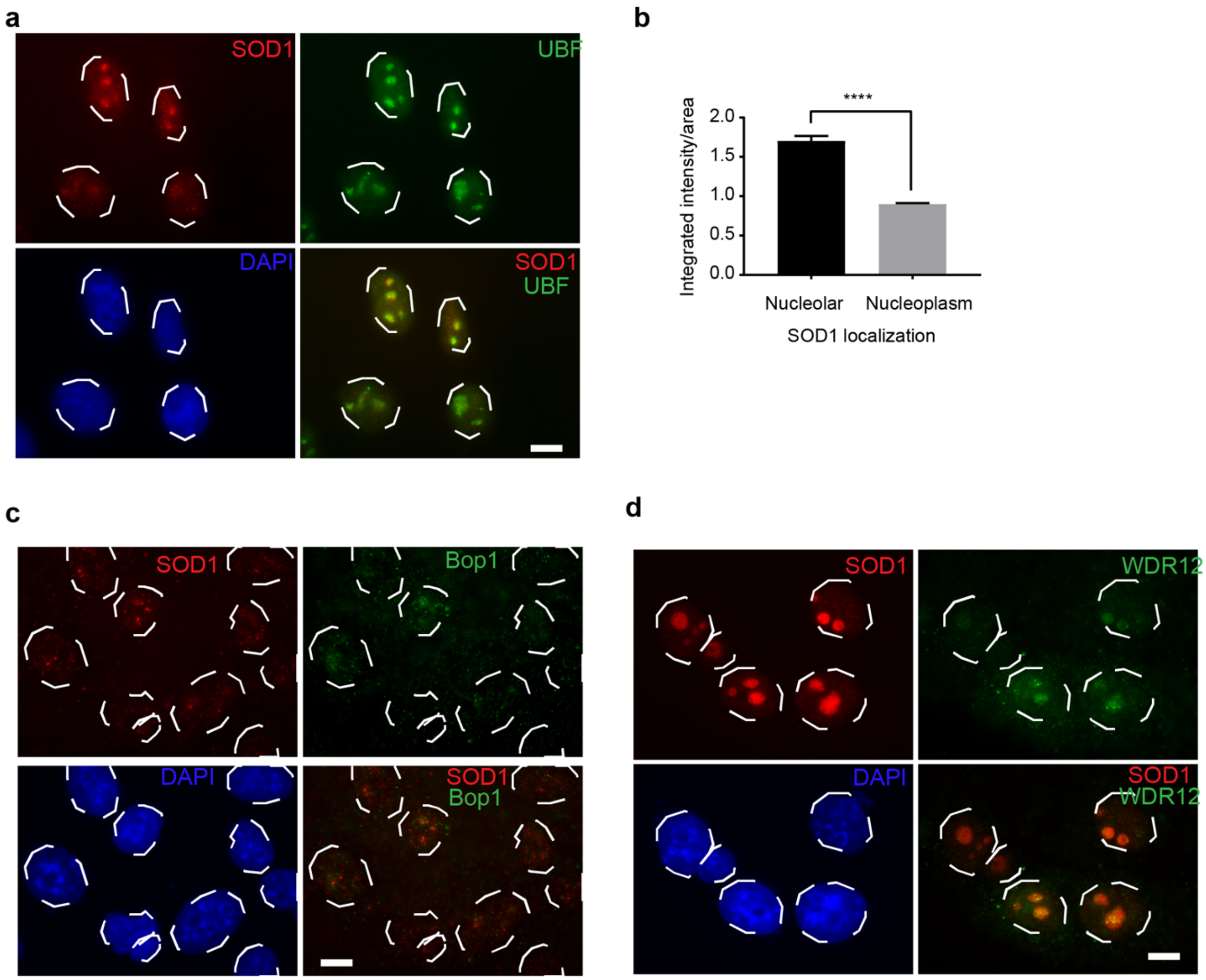

Supplementary Figure 8. SOD1 interacts with PeBoW complex and regulates pre-rRNA processing

a Flag-SOD1 is prominently localized in the nucleoli. *Sod1$^{Flox/Flox}$* KP NSCLC cells stably expressing Flag-SOD1 were treated with 4OHT and doxycycline (Dox) was added to the medium to induce expression of Flag-SOD1. Cells were fixed on day 5 post-treatment and analyzed by immunofluorescence analysis using antibodies against Flag (Red) and UBF (Green, nucloeli). Dashed lines demarcate the nucleus. Scale bars represent 10 μm. Data represent three independent experiments.

b-c Flag-SOD1 is co-localized with BOP1 (**b**) and WDR12 (**c**). *Sod1*$^{Flox/Flox}$ KP cells stably expressing Flag-SOD1 were treated with 4OHT and doxycycline to induce knockout of endogenous *Sod1* and expression of ectopic Flag-SOD1, respectively. IF analysis was performed with antibodies against Flag (Green) and BOP1 or WDR12 (Red). Dashed lines indicate the nucleus. Scale bar represents 10 μm. Data represent three independent experiments.

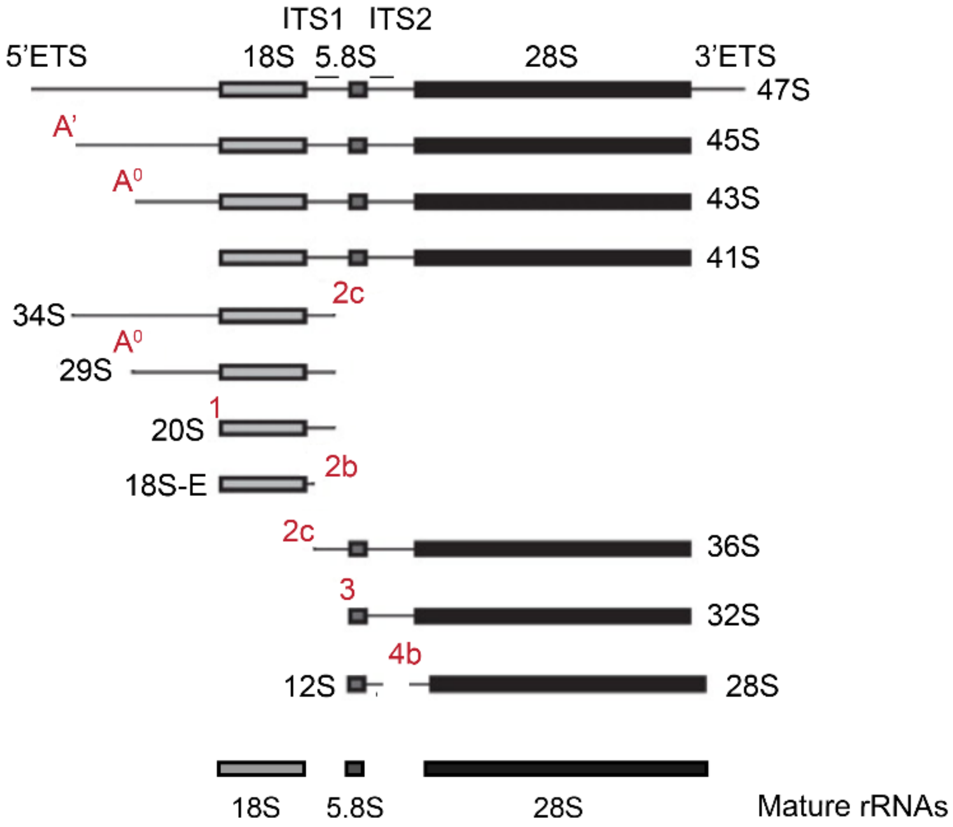

Supplementary Figure 9. Illustration of the structures of mouse 47S pre-rRNA and processing intermediates

Mouse 47S pre-rRNA are processed through multiple intermediates into mature 18S, 5.8S and 28S rRNA. The location of ITS1 and ITS2 is indicated, which was used as probe for Northern blot or IF. Processing sites are represented in red.

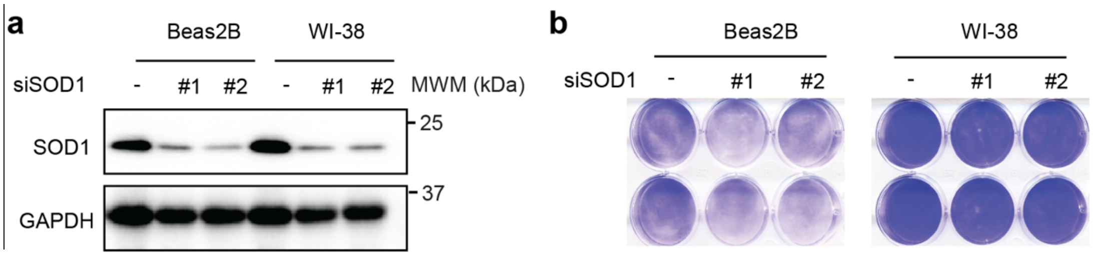

Supplementary Figure 10. SOD1 is not required for proliferation of normal human lung cells

Human normal lung fibroblasts (WI38) and human normal lung epithelial cells (Beas2B) transfected with human SOD1 siRNA or a control siRNA. The effect of SOD1 knockdown on SOD1 protein expression (**a**) and cell proliferation (**b**) were examined by immunoblot and plate growth assay, respectively. SOD1 protein expression data (**a**) present three independent experiments.

Supplementary Table 1. Oligoneucleotides

Gene	Strand	Sequence (5’->3’)
RT-qPCR primers		
Sod1	Forward	GGGTTCCACGTCCATCAGTAT
	Reverse	ACACATTGGCCACACCGTC
ActB	Forward	TGAGCGCAAGTACTCTGTGTGGAT
	Reverse	ACTCATCGTACTCCTGCTTGCTGA
Nqo1	Forward	TTCTCTGGCCGATTCAGAGT
	Reverse	GGCTGCTTGGAGCAAAATAG
Sod1 mutagenesis primers		
C147S	Forward	AAGTCGTTTGGCTAGTGGTGTAATTGG
	Reverse	CCAATTACACCACTAGCCAAACGACTT
G93A	Forward	ACAAAGATGCTGTGGCCGATGTG
	Reverse	CACATCGGCCACAGCATCTTTGT
NES	Forward	CCCATATCCTGGTCCTGCATTACGGTCTCTAAGAT ATCTAGACCCAGCTTTCTTG
	Reverse	CAGGATATGGGTCAATGCCAGACGCGTTTGGGCG ATCCCAATTACACC
NLS	Forward	CCAAAAAAGAAGAGAAAGGTACTCTAAGATATCTA GACCCAG
	Reverse	CCTTTCTCTTCTTTTTTGGACGCGTTTGGGCGATC CCAATTAC
Pre-rRNA probes		
mITS2		ACCCACCGCAGCGGGTGACGCGATTGATCG
hITS2		CAGCAGGTATGCCAGAAGCC

h5'ETS-01		GACAGGTCGCCAGAGGACAG
h28S-5'		GGTCGCCACGTCTGATC
h18S-5'		GAGCCATTCGCAGTTTCAC
RNA-FISH probes		
Human 5'ITS1-Cy3		CCTCGCCCTCCGGGCTCCGTTAATGATC
Mouse 5'ITS1-Cy3		ACGCCGCCGCTCCTCCACAGTCTCCCGTT
siRNA		
Si-hSOD1#1		5'-UCGUUUGGCUUGUGGUGUA-3'
Si-hSOD1#2		5'-UUAAUCCUCUAUCCAGAAA-3'
Si-mSOD1#1	Sigma	SASI_Mm01_00122733
Si-mSOD1#2	Sigma	SASI_Mm01_00122734
Control		5'-GTTCTCCGAACGTGTCAC-3'
