# Supplementary material for: SOD1 regulates ribosome biogenesis in KRAS mutant non-small cell lung cancer
Source: Nat Commun. 2021 Apr 15;12:2259. doi: 10.1038/s41467-021-22480-x (PMC8050259; doi:10.1038/s41467-021-22480-x)
Supplement: Supplementary file 2 — Reporting Summary [file 41467_2021_22480_MOESM2_ESM.pdf]

nature research

Corresponding author(s): X.F. Steven Zheng; Dimitri G Pestov

Last updated by author(s): 12/11/2020

Reporting Summary

Nature Research wishes to improve the reproducibility of the work that we publish. This form provides structure for consistency and transparency in reporting. For further information on Nature Research policies, see our Editorial Policies and the Editorial Policy Checklist.

Statistics

For all statistical analyses, confirm that the following items are present in the figure legend, table legend, main text, or Methods section.

n/a	Confirmed	
☐	☒	The exact sample size (*n*) for each experimental group/condition, given as a discrete number and unit of measurement
☐	☒	A statement on whether measurements were taken from distinct samples or whether the same sample was measured repeatedly
☐	☒	The statistical test(s) used AND whether they are one- or two-sided<br>*Only common tests should be described solely by name; describe more complex techniques in the Methods section.*
☒	☐	A description of all covariates tested
☒	☐	A description of any assumptions or corrections, such as tests of normality and adjustment for multiple comparisons
☐	☒	A full description of the statistical parameters including central tendency (e.g. means) or other basic estimates (e.g. regression coefficient) AND variation (e.g. standard deviation) or associated estimates of uncertainty (e.g. confidence intervals)
☐	☒	For null hypothesis testing, the test statistic (e.g. *F*, *t*, *r*) with confidence intervals, effect sizes, degrees of freedom and *P* value noted<br>*Give P values as exact values whenever suitable.*
☒	☐	For Bayesian analysis, information on the choice of priors and Markov chain Monte Carlo settings
☒	☐	For hierarchical and complex designs, identification of the appropriate level for tests and full reporting of outcomes
☒	☐	Estimates of effect sizes (e.g. Cohen's *d*, Pearson's *r*), indicating how they were calculated

Our web collection on statistics for biologists contains articles on many of the points above.

Software and code

Policy information about availability of computer code

Data collection	Fig. 5H, 6C - Amersham Typhoon control software ver. 2.0.0.6 (Cytiva), Fig. 7A - WinDaq ver. 3.76 (DATAQ Instruments, Inc).<br>Fig. 4E-I, Fig. 5A-F and Fig. S7B: Nikon A1R-Si Confocal Microscope System was used for confocal imaging and data collection.<br>Fig. S4 and Fig. S5- Infinite reader i-control software ver.1.12 (Tecan).
Data analysis	Analysis of Northerns: ImageQuant TL ver. 8.2 (Cytiva) and ImageQuant ver. 5.2 (Molecular Dynamics), image processing: Canvas ver. X.898 (ACD Systems, Inc), rRNA (Fig. 6D) and ROS levels quantification(Fig. S4 and Fig. S5): Prism 8.4.3 (GraphPad Software).

For manuscripts utilizing custom algorithms or software that are central to the research but not yet described in published literature, software must be made available to editors and reviewers. We strongly encourage code deposition in a community repository (e.g. GitHub). See the Nature Research guidelines for submitting code & software for further information.

Data

Policy information about availability of data

All manuscripts must include a data availability statement. This statement should provide the following information, where applicable:

- Accession codes, unique identifiers, or web links for publicly available datasets
- A list of figures that have associated raw data
- A description of any restrictions on data availability

The authors declare that the data supporting the findings of this study are available within the paper and supplementary information.
Fig. 7D-L, A transcriptome dataset available from The Cancer Genome Atlas (TCGA) database (https://www.cancer.gov/about-nci/organization/ccg/research/structural-genomics/tcga) was used to analyze SOD1 mRNA and ribosomal biogenesis gene expression.
Fig. 7D-The analysis of SOD1 mRNA expression in human primary lung adenocarcinoma (LUCA) and normal human lung tissues was performed using the website (http://ualcan.path.uab.edu/analysis.html).

Fig. 7L-The survival analysis of human LUCA tumors using SOD1 mRNA levels was performed using the GEPIA database (http://gepia.cancer-pku.cn/index.html).

Field-specific reporting

Please select the one below that is the best fit for your research. If you are not sure, read the appropriate sections before making your selection.

☒ Life sciences ☐ Behavioural & social sciences ☐ Ecological, evolutionary & environmental sciences

For a reference copy of the document with all sections, see nature.com/documents/nr-reporting-summary-flat.pdf

Life sciences study design

All studies must disclose on these points even when the disclosure is negative.

Sample size	Sample size was determined by power analysis of $\alpha = 0.5$, $\beta = 0.2$, and minimal detectable effect at 20%.
Data exclusions	No data exclusion performed.
Replication	All experiments have been repeated at least twice to ensure reproducibility.
Randomization	Mice and cell lines of distinct phenotype are assigned to respective group. Randomization is not applicable. All mice and cells were given the same treatment to ensure comparability.
Blinding	Blinding is not required as all data was collected by objective measurements.

Reporting for specific materials, systems and methods

We require information from authors about some types of materials, experimental systems and methods used in many studies. Here, indicate whether each material, system or method listed is relevant to your study. If you are not sure if a list item applies to your research, read the appropriate section before selecting a response.

Materials & experimental systems

n/a	Involved in the study	
☐	☒	Antibodies
☐	☒	Eukaryotic cell lines
☒	☐	Palaeontology and archaeology
☐	☒	Animals and other organisms
☒	☐	Human research participants
☒	☐	Clinical data
☒	☐	Dual use research of concern

Methods

n/a	Involved in the study	
☒	☐	ChIP-seq
☒	☐	Flow cytometry
☒	☐	MRI-based neuroimaging

Antibodies

Antibodies used

Immunohistochemistry:
name (provider, cat#,clone,dilution)
SOD1 (Abcam, cat# ab16831,Polyclonal,1:100)
Ki-67 (Thermo Scientific, cat# MA5-14520, SP6)
cleaved Caspase-3 (Cell Signaling, cat# 9664,5A1E)
phospho-histone H2A.X (Cell Signaling, cat# 9718,20E3)
ApopTag® Plus Peroxidase In Situ Apoptosis Kit (EMD Millipore, cat# S7101).
Biotinylated Goat Anti-Rat IgG Antibody, mouse adsorbed (vectorlabs, cat# BA-9401)

Western Blot:
HRP-Conjugated GAPDH Antibody (Proteintech, cat# HRP-60004,1:5000)
HRP-Conjugated Beta Actin Antibody (Proteintech, cat# HRP-66009,1:5000)
Phospho-p44/42 MAPK (Erk1/2) (Thr202/Tyr204) (D13.14.4E) XP® Rabbit mAb (Cell Signaling, cat# 4370T,D13.14.4E)
Phospho-EGF Receptor (Tyr1068) (D7A5) XP® Rabbit mAb (Cell Signaling, cat# 3777,D7A5)
Phospho-p38 MAPK (Thr180/Tyr182) (D3F9) XP® Rabbit mAb (Cell Signaling, cat# 4511,D3F9)
SOD1 (Santa Cruz Biotechnology, cat# sc-11407,FL-154,1:1000)
WDR12 Antibody, A302-651A (BETHYL, cat# A302-651A,Polyclonal,1:2000)
BOP1 Antibody, A302-149A (BETHYL, cat# A302-149A,Polyclonal,1:2000)
Exportin-1/CRM1 (D6V7N) Rabbit mAb (cellsignaling, cat# 46249,D6V7N,1:2000)
β-Actin (13E5) Rabbit mAb (Cell Signaling, cat# 4970,13E5,1:5000)

Immunofluorescence and PLA:
WDR12 Antibody, A302-651A (BETHYL, cat# A302-651A,Polyclonal,1:100)
BOP1 Antibody (Santa Cruz Biotechnology, cat# sc-390672,E-1,1:100)
Flag Rabbit mAb (Cell Signaling, 14793, 1:800)
NPM1 Monoclonal Antibody (FC-61991) (ThermoFisher, 32-5200, 1:200)

Validation

All antibodies are validated by the manufacturer according to the online information.
name provider cat# application website
SOD1 antibody Abcam cat# ab16831 WB, ICC/IF, IHC-P, IP, IHC-Fr https://www.abcam.com/superoxide-dismutase-1-antibody-ab16831.html
Ki-67 antibody Thermo Scientific cat# MA5-14520 FC,ICC,IF,IHC-F/P,WB,IHC,Misc,Neu https://www.thermofisher.com/antibody/product/Ki-67-Antibody-clone-SP6-Recombinant-Monoclonal/MA5-14520
cleaved Caspase-3 Cell Signaling cat# 9664 WB,IP,IHC,IF,F https://www.cellsignal.com/products/primary-antibodies/cleaved-caspase-3-asp175-5a1e-rabbit-mab/9664
phospho-histone H2A.X Cell Signaling cat# 9718 WB,IHC,IF,F https://www.cellsignal.com/products/primary-antibodies/phospho-histone-h2a-x-ser139-20e3-rabbit-mab/9718?site-search-type=Products&N=4294956287&Ntt=9718+&fromPage=plp&_requestid=1654275
Biotinylated Goat Anti-Rat IgG Antibody, mouse adsorbed vectorlabs cat# BA-9401 ELISA,ELISpot, ICC,IF,IHC,WB https://vectorlabs.com/biotinylated-goat-anti-rat-igg-antibody-mouse-adsorbed.html#additional
HRP-Conjugated GAPDH Antibody Proteintech cat# HRP-60004 WB, ELISA https://www.ptglab.com/products/GAPDH-Antibody-HRP-60004.htm
HRP-Conjugated Beta Actin Antibody Proteintech cat# HRP-66009 WB https://www.ptglab.com/products/beta-Actin-Antibody-HRP-66009.htm
Phospho-p44/42 MAPK (Erk1/2) (Thr202/Tyr204) (D13.14.4E) XP® Rabbit mAb cellsignaling cat# 4370T WB,IP,IHC,IF,F https://www.cellsignal.com/products/primary-antibodies/phospho-p44-42-mapk-erk1-2-thr202-tyr204-d13-14-4e-xp-rabbit-mab/4370?site-search-type=Products&N=4294956287&Ntt=4370t+&fromPage=plp&_requestid=1655193
Phospho-EGF Receptor (Tyr1068) (D7A5) XP® Rabbit mAb cellsignaling cat# 3777 WB,IHC,IF,F https://www.cellsignal.com/products/primary-antibodies/phospho-egf-receptor-tyr1068-d7a5-xp-rabbit-mab/3777
Phospho-p38 MAPK (Thr180/Tyr182) (D3F9) XP® Rabbit mAb cellsignaling cat# 4511 WB,IP,IHC,IF,F https://www.cellsignal.com/products/primary-antibodies/phospho-p38-mapk-thr180-tyr182-d3f9-xp-rabbit-mab/4511
SOD1 antibody Santa Cruz Biotechnology cat# sc-11407 WB, IP, IF, IHC(P) and ELISA https://www.scbt.com/p/sod-1-antibody-fl-154?requestFrom=search
WDR12 Antibody, A302-651A BETHYL cat# A302-651A WB https://www.bethyl.com/product/A302-651A?target=&referrer=search
BOP1 Antibody, A302-149A BETHYL cat# A302-149A WB, IP, IHC https://www.bethyl.com/product/A302-149A/BOP1+Antibody
Exportin-1/CRM1 (D6V7N) Rabbit mAb cellsignaling cat# 46249 WB, IP, IHC, IF https://www.cellsignal.com/products/primary-antibodies/exportin-1-crm1-d6v7n-rabbit-mab/46249
β-Actin (13E5) Rabbit mAb cellsignaling cat# 4970 https://www.cellsignal.com/products/primary-antibodies/b-actin-13e5-rabbit-mab/4970
BOP1 Antibody Santa Cruz Biotechnology cat# sc-390672 WB, IHC(P),ELISA https://www.scbt.com/p/bop1-antibody-e-1?requestFrom=search

Eukaryotic cell lines

Policy information about cell lines

Cell line source(s)

Mouse lung cancer cell lines were derived from SOD1fl/fl KP lung tumors in mice.
Mouse lung cancer cell lines(KP71-7, KP71-8) were derived from KP lung tumors in mice.
Mouse lung cancer cell lines(KL3-2, KP3-2) were derived from KL lung tumors in mice.
Human cell lines (Calu-1,H1155, A549, H460,H2030,Beas2B, WI-38) were purchased from American Type Culture Collection

Authentication

Mouse lung cancer cell lines were authenticated by PCR and immunoblot for SOD1, K-Ras, Lkb1 and TP53; ATCC uses morphology, karyotyping, and PCR based approaches to confirm the identity of human lung cancer cell lines Calu-1,H1155, A549, H460, H2030, Beas2B and WI-38.

Mycoplasma contamination

Tested negative by Sigma mycoplasma detection kit

Commonly misidentified lines
(See ICLAC register)

No commonly misidentified cell lines were used in the study.

Animals and other organisms

Policy information about studies involving animals; ARRIVE guidelines recommended for reporting animal research

Laboratory animals	All mice are of C57/B6 and 129 mixed background. UBC-Cre-ERT2 mice 21 (JAX stock #007001) and Sod1Flox/Flox mice (kindly provided by Dr. Holly Van Remmen) 20 were crossbred to generate UBC-Cre-ERT2-/+ Sod1Flox/Flox mice. UBC-Cre-ERT2-/+ Sod1Flox/Flox mice were further bred with KrasFSF-G12D/+ mice (JAX stock #008653) and Trp53frt/frt mice (JAX stock #017767) to generate UBC-Cre-ERT2-/+ Sod1+/+ KrasFSF-G12D/+ Trp53frt/frt mice and UBC-Cre-ERT2-/+ Sod1Flox/Flox KrasFSF-G12D/+ Trp53frt/frt mice. These strains used in this study were at the age ranging from 6 weeks to 28 weeks old. Both male and female were used.
Wild animals	No wild animals were used in the study.
Field-collected samples	No field-collected samples were used in the study.
Ethics oversight	Rutgers University IACUC

Note that full information on the approval of the study protocol must also be provided in the manuscript.
